# Cell-traversal protein for ookinetes and sporozoites (CelTOS) formulated with potent TLR adjuvants induces high-affinity antibodies that inhibit *Plasmodium falciparum* infection in *Anopheles stephensi*

**DOI:** 10.1186/s12936-019-2773-3

**Published:** 2019-04-24

**Authors:** Sakineh Pirahmadi, Sedigheh Zakeri, Akram A. Mehrizi, Navid D. Djadid, Abbas-Ali Raz, Jafar J. Sani, Ronak Abbasi, Zahra Ghorbanzadeh

**Affiliations:** Malaria and Vector Research Group (MVRG), Biotechnology Research Center (BRC), Pasteur Institute of Iran, Pasteur Avenue, P.O. Box 1316943551, Tehran, Iran

**Keywords:** *Plasmodium falciparum*, CelTOS, Transmission-reducing activity (TRA), CpG, Poly I:C

## Abstract

**Background:**

*Plasmodium falciparum* parasite is the most deadly species of human malaria, and the development of an effective vaccine that prevents *P. falciparum* infection and transmission is a key target for malarial elimination and eradication programmes. *P. falciparum* cell-traversal protein for ookinetes and sporozoites (PfCelTOS) is an advanced vaccine candidate. A comparative study was performed to characterize the immune responses in BALB/c mouse immunized with *Escherichia coli*-expressed recombinant PfCelTOS (rPfCelTOS) in toll-like receptor (TLR)-based adjuvants, CpG and Poly I:C alone or in combination (CpG + Poly I:C), followed by the assessment of transmission-reducing activity (TRA) of anti-rPfCelTOS antibodies obtained from different vaccine groups in *Anopheles stephensi.*

**Methods:**

The aim of the current work was achieved by head-to-head comparison of the vaccine groups using conventional and avidity enzyme-linked immunosorbent assay (ELISA), immunofluorescence test (IFAT), and standard membrane feeding assay (SMFA).

**Results:**

Comparing to rPfCelTOS alone, administration of rPfCelTOS with two distinct TLR-based adjuvants in vaccine mouse groups showed a significant increase in responses (antibody level, IgG subclass analysis, avidity, and Th1 cytokines) and was able to induce reasonable transmission-reducing activity. Also, comparable functional activity of anti-rPfCelTOS antibodies was found in group that received antigen in either CpG or Poly I:C (69.9%/20% and 73.5%/24.4%, respectively, reductions in intensity/prevalence). However, the vaccine group receiving rPfCelTOS in combination with CpG + Poly I:C showed a significant induction in antibody titers and inhibitory antibodies in oocysts development (78.3%/19.6% reductions in intensity/prevalence) in *An. stephensi*.

**Conclusions:**

A key finding in this investigation is that rPfCelTOS administered alone in BALB/c mouse is poorly immunogenic, with relatively low IgG level, avidity, inhibitory antibodies, and mixed Th1/Th2 responses. However, immunological characteristic (IgG level, cytophilic IgG2a and IgG2b, avidity, and Th1 cytokines) and TRA of anti-rPfCelTOS significantly enhanced in the presence of co-administration of TLR-based adjuvants, confirming that targeting TLRs would be an effective means for the enhancement of inducing TRA against rPfCelTOS.

**Electronic supplementary material:**

The online version of this article (10.1186/s12936-019-2773-3) contains supplementary material, which is available to authorized users.

## Background

The control of malaria mainly relies on anti-malarial drugs, rapid diagnosis, and vector control measures (such as insecticide-treated bed nets, or indoor insecticide spraying) and these interventions have reduced the mortality of malaria in recent years. However, due to increased resistance of mosquito vectors to insecticides and of parasites to available anti-malarial drugs, there are still 445,000 malaria-associated deaths worldwide every year, mostly because of *Plasmodium falciparum* [1]. Lessons from eradication of infectious diseases such as smallpox from the world [2, 3] and polio from the Western Hemisphere [4] have highlighted that to eliminate malaria, a vaccine would need to be included in the anti-malarial control tools. Such a vaccine would be expected to reduce the parasite reservoirs and interrupt malaria transmission [5].

In the new era of malaria vaccine development, a transmission-blocking vaccine (TBV) is considered an essential type of vaccine for the elimination of malaria [6, 7]. The aim of TBVs is to induce antibodies in human hosts against antigens expressed either during the sexual stage or antigens found in mosquito vectors. These antibodies must be able to inhibit parasite development in the *Anopheles* mosquito midgut, when they are ingested as a part of the blood meal with mature gametocyte stage parasites [8]. Although TBVs would not directly prevent infection in human host, they could help in elimination of the disease by preventing the transmission of infections [6, 7, 9, 10]. On this basis, the Malaria Eradication Research Agenda (malERA) Consultative Group has suggested the concept of “vaccine that interrupts malaria transmission (VIMT)” [6] in which this new proposed vaccine includes the classical TBVs, pre-erythrocytic and asexual blood-stage antigens that are able to protect immunized subjects from infection and also reducing parasite transmission. This type of malaria vaccines may possibly be an important tool for elimination programmes, as well as protecting against epidemics.

The malaria parasite infects both human and mosquito hosts and thus, an effective anti-malarial subunit vaccine may include antigens expressed in multiple stages of the life cycle. The cell-traversal protein for ookinetes and sporozoites (CelTOS) is a unique 25-kDa protein, which is critical for ookinete traversal of the mosquito midgut and for sporozoite infectivity of liver cells in human host [11]. Earlier works have demonstrated that irradiated sporozoite-immunized volunteers mount a strong immune response to CelTOS, and the immunization of mice with recombinant CelTOS (rCelTOS) induces both arms of the immune responses, as well as reduction in Plasmodium infection [12–15]. Thus, it is a promising transmission- and infection-blocking malaria subunit vaccine candidate [16, 17], that could induce broadly multiple immune responses to disrupt parasite infection in both human and mosquito hosts.

An ideal vaccine adjuvant is able to induce proper, potent and distinct types of specific immune responses, as well as to augment the quality, efficiency, and longevity of specific immune responses to a given antigens, with marginal toxicity to the immunized subjects. Among different vaccine adjuvants, toll-like receptor (TLR) agonists have shown promise in the clinical trials [18, 19]. One of the TLR adjuvants consists of synthetic oligodeoxynucleotides (ODNs) containing unmethylated CpG motifs (cytosine phosphate guanidine), which act as TLR-9 agonists [20, 21]. This adjuvant can increase the immunogenicity of recombinant protein antigens and improve/increase the function of professional antigen-presenting cells; as a result, inducing innate immunity allow to enhance antibody responses and to generate cellular (Th1 CD4+ T cell and CTL)-mediated responses [22]. Clinical trials have indicated that this adjuvant is safe when administered with target antigens in commercial Hepatitis B vaccine [23, 24], allergy [25], and cancer [26, 27]. Another Th1 potent TLR adjuvant is Poly I:C (polyinosinic:polycytidylic acid), a synthetic double-stranded RNA (dsRNA), that mimics viral RNAs and activates TLR-3 located within endosomes [28, 29]. The administration of this adjuvant activates dendritic cells that quickly produce IL-12 and type I IFN, both play a crucial role in the induction of Th1 responses [30–32]. This adjuvant is one of the most important TLR-3 agonists tested against diseases such as HIV [33, 34], dengue [35], malaria [36], and cancer [37, 38].

The recently updated Malaria Vaccine Technology Road map to 2030 [39] has suggested that a highly effective vaccine (as a new strategy) is required to prevent disease transmission. On this basis, the idea that CelTOS is one of the important proteins for the traversal of the *Plasmodium* species in both human and female mosquito hosts has encouraged further evaluation of whether specific immune responses against rPfCelTOS formulated in different potent vaccine adjuvants could inhibit the infection in *Anopheles stephensi*, as the mosquito host. Therefore, the current study aimed to assess the immune responses to expressed rPfCelTOS protein in *Escherichia coli*. It was hypothesized that TLRs-based human-use compatible adjuvants, CpG ODN and Poly I:C with rPfCelTOS antigen (the adjuvanted vaccine groups), would enhance the immunogenicity of the recombinant antigen, as well as the avidity and functional transmission-reducing activity (TRA) of anti-rPfCelTOS antibodies compared to antigen alone (the non-adjuvanted group) in immunized BALB/c mouse. This aim was achieved by head-to-head comparison of the experimental mouse groups using conventional and avidity enzyme-linked immunosorbent assay (ELISA) along with standard membrane feeding assay (SMFA). In addition, the second hypothesis was that whether in comparing to adjuvant alone, co-administration of both adjuvants with rPfCelTOS would enhance better acquired immune responses in mice and also TRA in the *An. stephensi* mosquito host. This work is an important step towards the development of a PfCelTOS-based vaccine in humans.

## Methods

### Expression and purification of rPfCelTOS protein

The sequences of *pfceltos* gene representing amino acids 25–182 were cloned into pET23a-plasmid and expressed in *E. coli* BL21 (DE3), as described previously [40]. In brief, the *E. coli* BL21-pET23a-PfCelTOS-clone was grown in Terrific Broth (TB) that contains 100 μg/mL ampicillin and at OD_600nm_ 0.6–0.8, the PfCelTOS expression was induced by isopropyl-beta-d-1-thiogalactopyranoside (IPTG, Thermo Scientific, Waltham, Massachusetts, USA), and the cells were grown for 16 h. The rPfCelTOS was purified with the Ni–NTA agarose (Qiagen, Hilden, Germany) and desalted using Econo-Pac 10 DG columns (BioRad, Hercules, CA, USA) according to the manual described by the manufacturer. In the next step, Bradford protein assay was used to measure the concentration of rPfCelTOS protein using a spectrophotometer (DeNovix, Wilmington, DE, USA) at OD_595nm_. Under reducing (with 1% sodium dodecyl sulfate [SDS] and 2% β-mercaptoethanol [2ME]) condition by 15% SDS–polyacrylamide gel electrophoresis (SDS-PAGE) the desalted protein was analysed. Western blot assay was performed using anti-His antibody (Penta-His Antibody; Qiagen, Germany) and sera from *P. falciparum*-infected patients [40] to confirm the purity of the rPfCelTOS. Finally, the level of *E. coli* endotoxin was estimated using the LAL kit (Lonza, Walkersville, MD, USA). This evaluation was carried out in the Quality Control Unit of the Recombinant Protein Production Complex of Pasteur Institute of Iran in Karaj.

### Immunization of BALB⁄c mice with rPfCelTOS

To produce polyclonal antibodies against rPfCelTOS, inbred female BALB⁄c mice (6- to 8-week-old) were received from the Laboratory Animal Science Department of Pasteur Institute of Iran. The mice were housed in the animal care unit and allowed to adapt for 1 week before the experiment. The animal procedures were approved by the Committee of Animal Ethics of Pasteur Institute of Iran (IR.PII.REC.1395.36). All the BALB⁄c mice (n = 160) were randomly distributed into 10 groups (n = 16/group, Table 1) and were immunized subcutaneously at the base of tail with the rPfCelTOS (10 μg/mouse/prime and 5 μg/mouse/boost) alone (non-adjuvanted vaccine group) or formulated in CpG (10 μg/mouse, vaccine grade type, InvivoGen, San Diego, CA, USA), Poly I:C (10 μg/mouse, vaccine grade type, InvivoGen, San Diego, CA, USA), and in combination with both CpG (5 μg/mouse) plus Poly I:C (5 μg/mouse) adjuvants (adjuvanted vaccine groups), three times at 14-day intervals. The mice were also injected with rPfCelTOS plus complete Freund’s adjuvant (CFA) as reference adjuvant (in prime) or incomplete Freund’s adjuvant (IFA, in the first and second boosts), both from Sigma-Aldrich Co., St. Louis, MO, (USA), with 1:1 volume/volume ratio (Table 1). The mice in the negative control groups were similarly received sterile PBS (PBS 1×, pH 7.4), CpG, Poly I:C, CpG + Poly I:C, and CFA/IFA (Table 1). For the evaluation of anti-rPfCelTOS antibody responses, sera were obtained from immunized mice prior to the first injection [as pre-immune sera/normal mouse sera (NMS)] and also on days 10, 24, and 38 after the primary immunization (Table 1). The collected sera samples were kept at − 20 °C.

**Table 1:** Mice immunization and bleeding strategies

	Immunization formulation	Antigen/adjuvant(s)				
		rPfCelTOS (µg/mouse)		CpG (µg/mouse)	Poly I:C (µg/mouse)	CFA/IFA (µL)
		Prime	Boosts			
*Vaccine groups*						
1	rPfCelTOS (Ag)	10	5	–	–	–
2	Ag/CpG	10	5	10	–	–
3	Ag/Poly I:C	10	5	–	10	–
4	Ag/CpG + Poly I:C	10	5	5	5	–
5	Ag/CFA/IFA	10	5	–	–	100
*Control groups*						
6	CpG	–	–	10	–	–
7	Poly I:C	–	–	–	10	–
8	CpG + Poly I:C	–	–	5	5	–
9	CFA/IFA	–	–	–	–	100
10	PBS 1×	–	–	–	–	–

Mice received 200 μL rPfCelTOS (10 μg at prime and 5 μg at boost) alone (non-adjuvanted vaccine group) and in the presence of CpG (10 μg/mouse, vaccine grade type, InvivoGen, San Diego, CA, USA), Poly I:C (10 μg/mouse, vaccine grade type, InvivoGen, San Diego, CA, USA), and combination of both CpG (5 μg/mouse) and Poly I:C (5 μg/mouse) adjuvants (adjuvanted vaccine groups) subcutaneously. Also, the mice were immunized with rPfCelTOS emulsified in CFA (complete Freund’s adjuvant) as reference adjuvant (the first dose) or IFA (incomplete Freund’s adjuvant; subsequent doses), with 1:1 volume/volume ratio. Control mice immunized with sterile PBS 1× (pH 7.4), CFA/IFA (as the reference adjuvant), CpG, Poly I:C, and CpG + Poly I:C. Sera samples were collected from the tail vein 10 days after each immunization (days 10, 24, and 38 after the first immunization)

*Ag* rPfCelTOS antigen, *CFA* complete Freund’s adjuvant, *IFA* incomplete Freund’s adjuvant, *CpG* synthetic oligodeoxynucleotides (ODNs) containing unmethylated CpG motifs, *Poly I:C* polyinosinic:polycytidylic acid

### ELISA-based measurement of antibody responses to rPfCelTOS

Anti-rPfCelTOS antibodies were evaluated in immunized mouse sera at 10, 24, and 38 days of the primary immunization using ELISA, as reported earlier [41] with minor modifications. In brief, diluted rPfCelTOS protein (20 ng/well) in a coating buffer was added into MaxiSorp flat-bottom 96-well ELISA plates (Jet Biofil, Guangzhou, China) and incubated at 4 °C overnight. After washing the plates three times with PBS 1× containing 0.05% Tween 20 (PBS-T), the wells were blocked with 1% BSA (Roche, Switzerland) at room temperature (RT) for 2 h. Then, 1:200 diluted sera samples in PBS-T with 0.5% BSA were used and the plates were incubated for 90 min. The plates were washed with PBS-T and then incubated with 100 μL of goat anti-mouse IgG antibodies conjugated with horse-radish peroxidase (HRP; 1:25 000; Sigma-Aldrich Co.) at RT for 1 h. Anti-rPfCelTOS IgG was detected using *o*-phenylenediamine (OPD)⁄H_2_O_2_ (Sigma-Aldrich Co.) as a substrate. The reaction was stopped with 2 N H_2_SO_4_, and OD_490nm_ was measured using an ELISA microplate reader (BioTek, Winooski, VT, USA). The cut-off values were estimated from the average of the 20 NMS plus three standard deviations (SD). Further, the anti-rPfCelTOS IgG subclasses were evaluated by the ELISA as described above; however, 1: 1000 dilution of the secondary antibodies that were specific to mouse IgG1, IgG2a, IgG2b, and IgG3 (Sigma-Aldrich Co.) antibodies were used. Subsequently, the plates were incubated with 1: 10 000 dilution of anti-goat IgG HRP (Sigma-Aldrich Co.) at RT for 1 h, as mentioned above.

### Anti-rPfCelTOS antibody avidity

Anti-rPfCelTOS IgG and its subclasses (IgG2a and IgG2b) antibody avidity assays were performed as reported before [42] with some modifications. In brief, two MaxiSorp plates (Jet Biofil) were coated with 20 ng/well rPfCelTOS protein and then blocked with 1% BSA for 1 h. Then, the plates were incubated with 1: 200 diluation of mouse sera at RT for 90 min. The plates were washed three times with either PBS-T or dissociation buffer containing PBS-T-urea (8 M) with vigorous shaking. Afterward, the two plates were washed with PBS-T buffer and then incubated with secondary antibody. The development of enzyme reaction was performed as described above for ELISA experiment. The avidity index (AI) was expressed by the ratio between urea-treated to non-treated samples multiplied by 100.

### Lymphocyte proliferation assay and detection of cytokines by ELISA

The lymphocyte proliferation of mouse was performed by 3-(4,5-dimethylthiazol-2-yl)-2,5-diphenyltetrazolium bromide (MTT) dye assay. In detail, four mice from each vaccinated and control groups were euthanized under sterile conditions. After removal of the spleens, the preparation of single-cell suspensions was made in RPMI 1640 medium (Gibco, Invitrogen, Scotland, UK) and red blood cells were removed with an ammonium chloride potassium lysis buffer (pH 7.2). After washing step, the cells were re-suspended in RPMI 1640 medium containing 5% fetal calf serum (FCS, Sigma-Aldrich Co.), 10 mM HEPES (Sigma-Aldrich Co.), 2.3 × 10^−2^ mM 2-mercaptoethanol (2-ME), and 100 U–100 μg/mL penicillin–streptomycin. Trypan blue dye exclusion was used to confirm the cell viability. Afterward, 100 μL of cell suspension (2 × 10^6^ cell/mL) were plated in 96-well tissue culture plate (Orange Scientific, EU, Belgium) in the presence of rPfCelTOS (10 μg/mL), concanavalin A (ConA; 5 μg/mL), and medium alone (negative control). After 48 h, the supernatants were discarded, and cell proliferation was estimated by MTT assay as described before [41]. Stimulation index (SI) was estimated by the ratio of the mean OD_550nm_ of the antigen stimulated spleen cells divided by the mean OD_550nm_ of the unstimulated cells.

In the supernatants of stimulated splenocytes of vaccinated mice with rPfCelTOS the cytokine profiles were analysed using mouse cytokine ELISA kits (R&D system, Minneapolis, USA). The splenocytes of each mouse group (n = 4) were cultured as described above. The culture supernatants were obtained after 24 and 48 h for IL-4 and 72 h for IL-10, TNF, and IFN-γ. The concentration of cytokines was calculated based on the standard curves performed in parallel with the known concentrations of recombinant mouse IL-4, IL-10, TNF, and IFN-γ for each experiment. The mean of concentration ± SD was recorded for each set of tested samples.

### Inhibition of *Plasmodium falciparum* infection in *Anopheles stephensi* by standard membrane feeding assay

The functional activity of anti-rPfCelTOS IgG antibodies in reducing transmission of *P. falciparum* strain NF54 (a kind gift from Dr. T. Fandeur, Institut Pasteur, Paris) in female *An. stephensi* (mysorensis strain) was assessed by SMFA. The gametocyte culture was carried out as described earlier [43], and cultures were monitored microscopically daily. Different stages of gametocytes were classified according to the Carter and Miller guidelines [44]. *P. falciparum* (NF54) mature gametocytes (0.15%–3%) were harvested 12 days after initiation [43] with 1 male: 2–4 females ratio. *Anopheles stephensi* mysorensis colony is being maintained at the National Insectarium of Pasteur Institute of Iran (Karaj, Iran). For the SMFA, *An. stephensi* (4–5 days old, n = 50 per cup) were put in pint-size ice-cream cups 1 day before infectious blood feeding and starved overnight. For feeding female *An. stephensi* via a parafilm membrane, mature stage V *P. falciparum* NF54 gametocytes (with 1–3 exflagellating centres per 40× field) were mixed with washed human O^+^ blood group at 50% haematocrit in pre-warmed human AB^+^ serum. Then an equal amount (human sera) of either pooled sera from negative control groups [(NMS, non-adjuvanted control group), CpG, Poly I:C, and CpG + Poly I:C (adjuvanted control groups)] or from different vaccinated groups was added to the mixture (groups 1–4, Table 1). Female mosquitoes were allowed to blood-feed for 20 min, and the fully engorged *Anopheles* mosquitoes were selected and maintained under insectary conditions of 26 °C temperature, and 80% humidity. Engorged mosquitoes were continuously supplied with 10% sugar on water-soaked cotton, and on days 9–10, the infection of midguts was assessed from surviving mosquitoes using 0.2% mercurochrome in PBS 1×. The stained oocysts were counted under a light microscope, and the percentage of inhibition in infection intensity and the prevalence were estimated relative to the control group. Two independent replicate experiments were performed, and the percentage of reduction in mean oocyst intensity was estimated as follows: 100 × {1 − (mean number of oocysts in the test group/mean number of oocysts in the control groups)}. Similarly, the percentage inhibition of oocyst prevalence was calculated based on the following formula: 100 × {1 − (proportion of mosquitoes with any oocysts in the experimental group/proportion of mosquitoes with any oocysts in the control group)}.

### Statistical analysis

A database was generated with IBM SPSS 21.0 for Windows (Armonk, NY: IBM Corp, USA). The differences in the antibody levels and cytokine responses in vaccine mouse groups were analysed by one-way ANOVA followed by Tukey’s honestly significant difference (HSD) post hoc test*. P *< 0.05 was considered as significantly (*P < 0.05, **P < 0.001, ***P < 0.0001). In addition, to compare the antibody level in each group on days 10, 24, and 38 of the primary immunization paired sample *t*-test was performed, and *P *< 0.05 were considered statistically significant. For SMFA, significance was assessed using Mann–Whitney *U* (to examine differences in intensity) and Fisher’s exact (to examine differences in prevalence) tests.

## Results

### Expression and purification of rPfCelTOS

The rPfCelTOS was successfully cloned and expressed in *E. coli* BL21-pET23a expression system, as shown in an earlier work [40]. The rPfCelTOS expression was achieved in TB medium, 16 h after induction with 0.5 mM IPTG. SDS-PAGE and Western blot analysis were used to confirm the purity of rPfCelTOS using anti-His antibody, sera from *P. falciparum*-infected patients (positive control, n = 10), and sera from healthy human (negative control, n = 20), and the result revealed a ~ 20 kDa single band [40]. Additionally, Western blot analysis of pooled sera from the vaccinated mice with rPfCelTOS (groups 1–5) demonstrated that the purified rPfCelTOS was recognized by anti-rPfCelTOS IgG (Additional file 1: Figure S1). Also, anti-rPfCelTOS IgG antibodies identified the native CelTOS protein on the surface of *P. falciparum* sporozoite (Multi-spot slides obtained as a kind gift from Dr. G. Snounou), (Additional file 1: Figure S2). However, the native PfCelTOS protein expressed by parasite was not recognized by the sera obtained from the control mouse groups (Table 1), confirming rPfCelTOS-specific responses. The endotoxin concentration of the purified rPfCelTOS using the LAL chromogenic kit demonstrated an acceptable amount of 0.1 EU/mL (the injection of 200 μL rPfCelTOS/mouse provides 0.02 EU of endotoxin into each mouse).

### Anti-rPfCelTOS IgG antibody responses

**Fig. 1:**
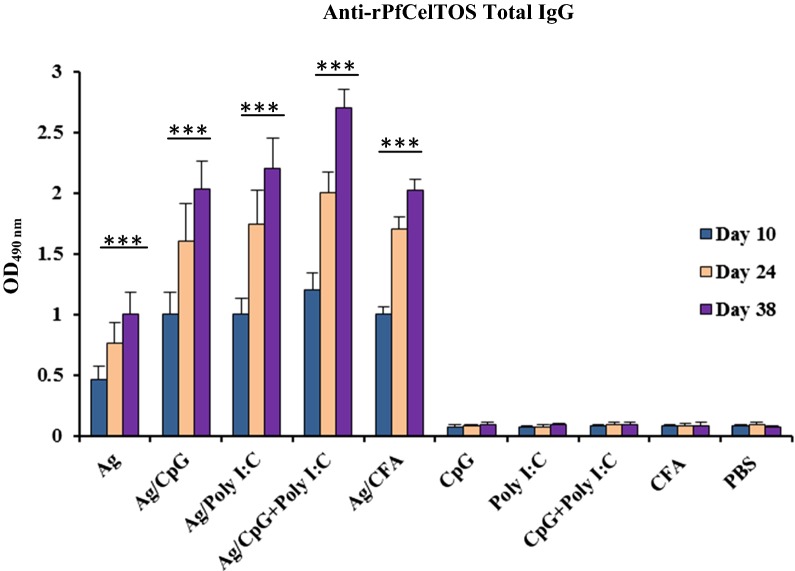
Anti-rPfCelTOS IgG antibody levels in vaccine mouse sera at different time points after immunization by ELISA. Anti-rPfCelTOS IgG antibody levels were compared between the collected sera from immunized mice on days 10, 24 (10 days after the first boost), and 38 (10 days after the second boost) of the first immunization. The sera samples were incubated in duplicate wells with 1:200 diluted sera for 90 min. There was a significant difference in total IgG antibody levels between different immunization time points in vaccine groups (paired sample *t*-test, *P* < 0.0001). The highest level of anti-rPfCelTOS IgG antibody was identified in the groups that received rPfCelTOS in combination with CpG + Poly I:C, (*P* < 0.0001, one-way ANOVA). Data represents means from three separate ELISA experiments. The bars and error bars show the mean OD_490nm_ and SD of 16 individual mice in each group, respectively. The ELISA cut-offs were calculated as the mean OD_490nm_ of NMS (as the negative controls, n = 20) plus 3 SD. The cut-offs for total IgG on days 10, 24, and 38 were almost 0.109, 0.099 and 0.101, respectively. ***P < 0.0001. *Ag* rPfCelTOS antigen, *NMS* normal mouse sera

**Fig. 2:**
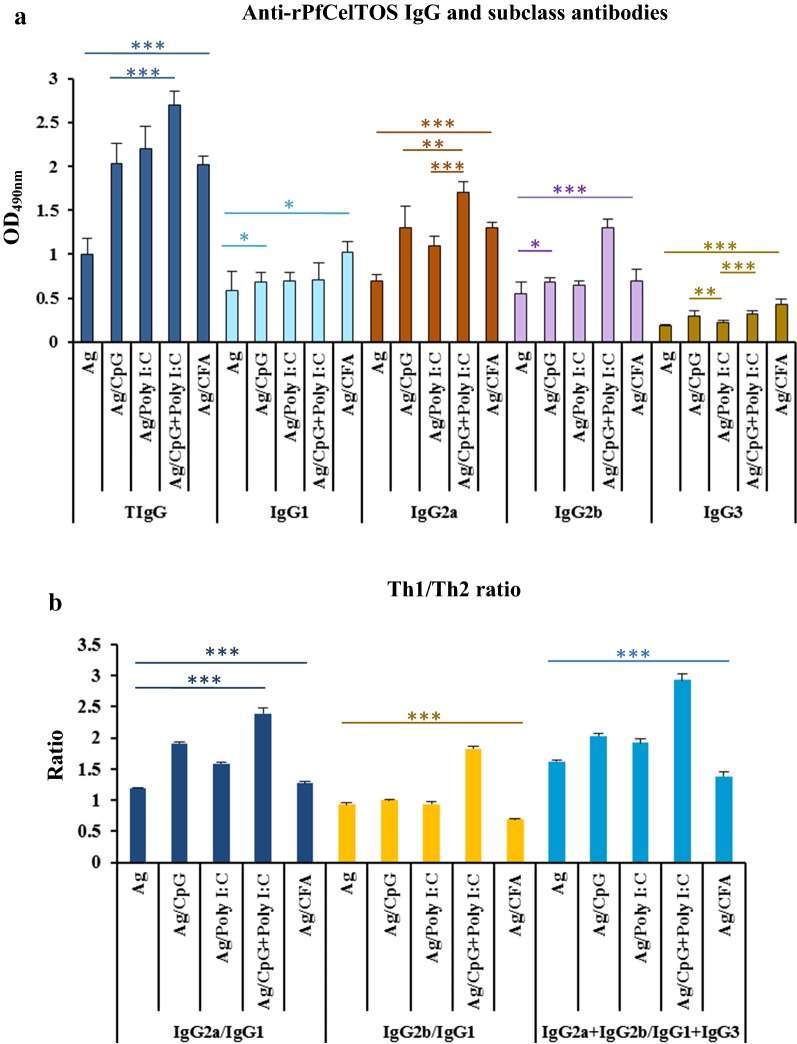
Assessment and comparison of IgG and IgG subclass antibodies to rPfCelTOS in the sera of vaccinated mice by ELISA (**a**) and Th1/Th2 responses ratios (**b**). Groups of 6- to 8-week-old female BALB⁄c mice were immunized subcutaneously with rPfCelTOS alone or formulated in different adjuvants: CpG, Poly I:C, CpG + Poly I:C, and CFA/IFA (as the reference adjuvant). Anti-rPfCelTOS IgG and its subclasses were assessed in individual immunized mice on day 38 of the first immunization (**a**). IgG2a was the predominant isotype in all vaccine groups (1–5). The highest levels of IgG2a and IgG2b subclasses were detected in the group received rPfCelTOS in combination with CpG + Poly I:C (*P *< 0.0001, one-way ANOVA). The bars and error bars show the mean OD_490nm_ and SD of 16 individual mice in each group, respectively. The ELISA cut-offs were calculated as the mean OD_490nm_ of NMS (as the negative controls, n = 20) plus 3SD. The cut-off values for total IgG, IgG1, IgG2a, IgG2b, and IgG3 antibodies were 0.101, 0.064, 0.072, 0.093, and 0.091, respectively. The Th1/Th2 ratio (IgG2a/IgG1, IgG2b/IgG1 and IgG2a + IgG2b/IgG1 + IgG3) was performed in the sera of immunized mice on day 38 after the first immunization (**b**) and among all vaccinated groups; the highest and a significant difference in all ratios was observed in group 4, rPfCelTOS/CpG + Poly I:C (*P *< 0.0001, one-way ANOVA). Data was analysed using one-way ANOVA followed by Tukey’s HSD post hoc test. *P < 0.05, **P < 0.001, ***P < 0.0001. *Ag* rPfCelTOS antigen, *TIgG* total IgG, *NMS* normal mouse sera

Comparing the antibody responses to rPfCelTOS antigen between the prime (day 10) and the two boosts (days 24 and 38) in all vaccine groups (1–5; Table 1) demonstrated a significant increase in the level of anti-rPfCelTOS IgG antibodies (Fig. 1) on days 24 and 38 relative to day 10 of the first immunization (*P *< 0.0001, paired sample *t*-test). There was no detectable specific anti-rPfCelTOS IgG antibodies in the control mouse groups that received adjuvant(s) without antigen (Additional file 1: Figure S3). In the adjuvanted vaccine groups, the highest level of specific anti-rPfCelTOS IgG antibodies was observed in the mice group 4 that received antigen formulated in CpG + Poly I:C (mean OD_490nm_: 2.7; *P *< 0.0001, one-way ANOVA; Fig. 2a and Additional file 2: Table S1). In addition, the multiple comparisons among all the vaccine groups (1–5) showed the statistically significant increase in the level of antibodies in the adjuvanted (2–5) rather than non-adjuvanted (1) group (*P *< 0.0001, Tukey’s HSD post hoc test; Fig. 2a and Additional file 2: Table S1).

### Anti-rPfCelTOS IgG isotypes

Analysis of distribution frequencies of specific anti-rPfCelTOS IgG isotypes in all the vaccine groups (1–5) on day 38 after the primary immunization revealed a bias toward IgG2a > IgG2b > IgG1 > IgG3 antibody subclasses (Fig. 2a). The highest level of anti-rPfCelTOS IgG2a and IgG2b was detected in the vaccine group 4 receiving rPfCelTOS in combination with CpG + Poly I:C adjuvants (mean OD_490nm_: 1.7 and 1.3, respectively; Fig. 2a). Comparison between non-adjuvanted (1) and adjuvanted (2–5) vaccine groups revealed a significant increase in the level of specific anti-rPfCelTOS IgG2a antibodies in the adjuvanted vaccine groups (*P *< 0.0001, one-way ANOVA; Fig. 2a and Additional file 2: Table S1). In addition, among adjuvanted vaccine groups, a statistically significant difference was found in the levels of IgG2a and IgG2b antibodies between the group 4 that received rPfCelTOS in combination with CpG + Poly I:C and groups receiving CpG, Poly I:C, or CFA/IFA adjuvants alone (*P *< 0.001, Tukey’s HSD post hoc test; Fig. 2a and Additional file 2: Table S1). Multiple comparisons of the adjuvanted vaccine groups showed no significant difference in the level of anti-rPfCelTOS IgG2a and IgG2b antibody subclasses among the vaccine group 2 (rPfCelTOS/CpG), 3 (rPfCelTOS/Poly I:C), and 5 (rPfCelTOS/CFA/IFA) (*P *> 0.05, Tukey’s HSD post hoc test; Fig. 2a and Additional file 2: Table S1). Regarding anti-PfCelTOS IgG1 and IgG3 isotypes, the highest and the most significant level of these IgG isotypes was found among the mouse group 5 that received antigen in CFA/IFA (mean OD_490nm_: 1.02 and 0.43, respectively; *P *< 0.05, one-way ANOVA; Fig. 2a and Additional file 2: Table S1). Furthermore, multiple comparisons of the adjuvanted vaccine groups (2–5) showed a statistically significant difference in the level of anti-rPfCelTOS IgG3 antibodies in the vaccine group 4 receiving rPfCelTOS in combination with CpG + Poly I:C adjuvants compared to the immunized mouse groups 3 (rPfCelTOS/Poly I:C) and 5 (rPfCelTOS/CFA/IFA) (*P *< 0.0001, Tukey’s HSD post hoc test; Fig. 2a and Additional file 2: Table S1).

Multiple comparisons of the Th1/Th2 response ratio among the vaccine mouse groups (1–5) showed the highest and significant IgG2a/IgG1 (2.39), IgG2b/IgG1 (1.83), and IgG2a + IgG2b/IgG1 + IgG3 (2.93) ratios among the mouse group 4 receiving rPfCelTOS formulated with CpG + Poly I:C adjuvants (*P *< 0.0001, Tukey’s HSD post hoc test; Fig. 2b and Additional file 2: Table S1). The lowest IgG2a/IgG1 (1.19) ratio was observed in the non-adjuvanted vaccine group 1 that received rPfCelTOS antigen alone (Fig. 2b). However, the lowest and the significant IgG2b/IgG1 (0.69) and IgG2a + IgG2b/IgG1 + IgG3 (1.38) ratios were found in the adjuvanted vaccine group 5 receiving rPfCelTOS antigen in the reference adjuvant CFA/IFA (*P *< 0.05, Tukey’s HSD post hoc test; Fig. 2b and Additional file 2: Table S1). The IgG2b/IgG1 ratio was not statistically significant in groups received antigen alone and groups 2 and 3 receiving antigen formulated in CpG or Poly I:C adjuvant individually, respectively (*P *> 0.05, Tukey’s HSD post hoc test; Fig. 2b and Additional file 2: Table S1). Besides, no significant difference was detected in the IgG2b/IgG1 and IgG2a + IgG2b/IgG1 + IgG3 ratios between the adjuvanted vaccine groups 2 and 3 that received rPfCelTOS in CpG and Poly I:C adjuvants alone, respectively (*P *> 0.05, Tukey’s HSD post hoc test; Fig. 2b and Additional file 2: Table S1).

### Anti-rPfCelTOS IgG and subclasses avidity

**Fig. 3:**
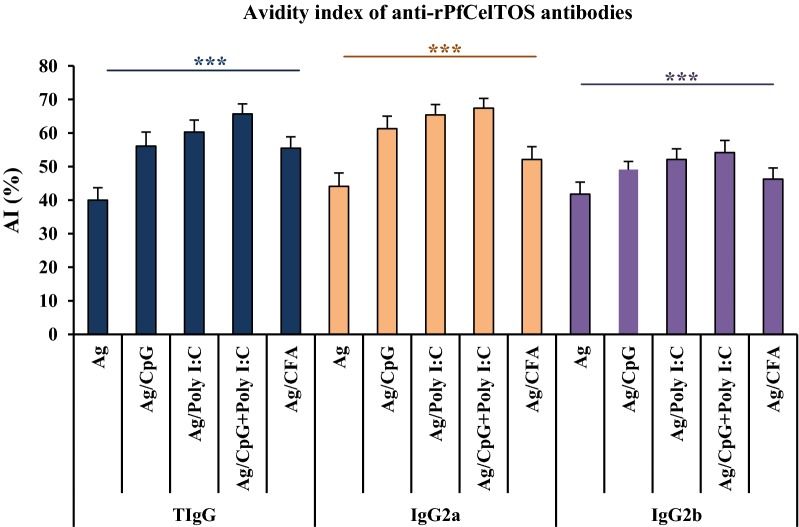
Evaluation of anti-rPfCelTOS IgG, IgG2a, and IgG2b antibody avidity in immunized mice on day 38 of the first immunization by ELISA. A significant difference was observed in the avidity index (AI) of IgG, IgG2a and IgG2b antibodies between mouse group 4 (rPfCelTOS/CpG + Poly I:C) and groups 1 (rPfCelTOS alone), 2 (rPfCelTOS/CpG), and 5 (rPfCelTOS/CFA/IFA) (*P *< 0.05, one-way ANOVA). AI values ≤ 30%, between 30 and 50%, and > 50% were considered as low-, intermediate- and high-avidity anti-rPfCelTOS-specific antibodies, respectively. The bars and error bars show the mean AI and standard deviation (SD) in each mouse group (n = 16), respectively. *P < 0.0001. *Ag* rPfCelTOS antigen, *TIgG* Total IgG

High-avidity IgG and IgG2a antibodies were induced in the adjuvanted vaccine groups (2–4) receiving recombinant antigen formulated in different adjuvants: CpG, Poly I:C, and in combination with CpG + Poly I:C on days 38 after the primary immunization (Fig. 3). However, intermediate avidity for IgG and IgG2a antibodies was detected in mice receiving rPfCelTOS antigen alone (Fig. 3). Regarding IgG2b, the high-avidity antibody was observed in the mouse groups 3 (rPfCelTOS/Poly I:C) and 4 (rPfCelTOS/CpG + Poly I:C), whereas intermediate-avidity IgG2b antibodies were found among the vaccine groups 1, 2, and 5 (Fig. 3). Among all the vaccinated mouse groups (1–5), a higher and a statistically significant increase in the high-avidity of IgG, IgG2a and IgG2b antibodies was detected in the group 4 (rPfCelTOS/CpG + Poly I:C) compared with mouse groups 1 (rPfCelTOS alone), 2 (rPfCelTOS/CpG) and 5 (rPfCelTOS/CFA/IFA) (*P *< 0.05, Tukey’s HSD post hoc test; Fig. 3 and Additional file 2: Table S1).

### Cellular immune responses

**Fig. 4:**
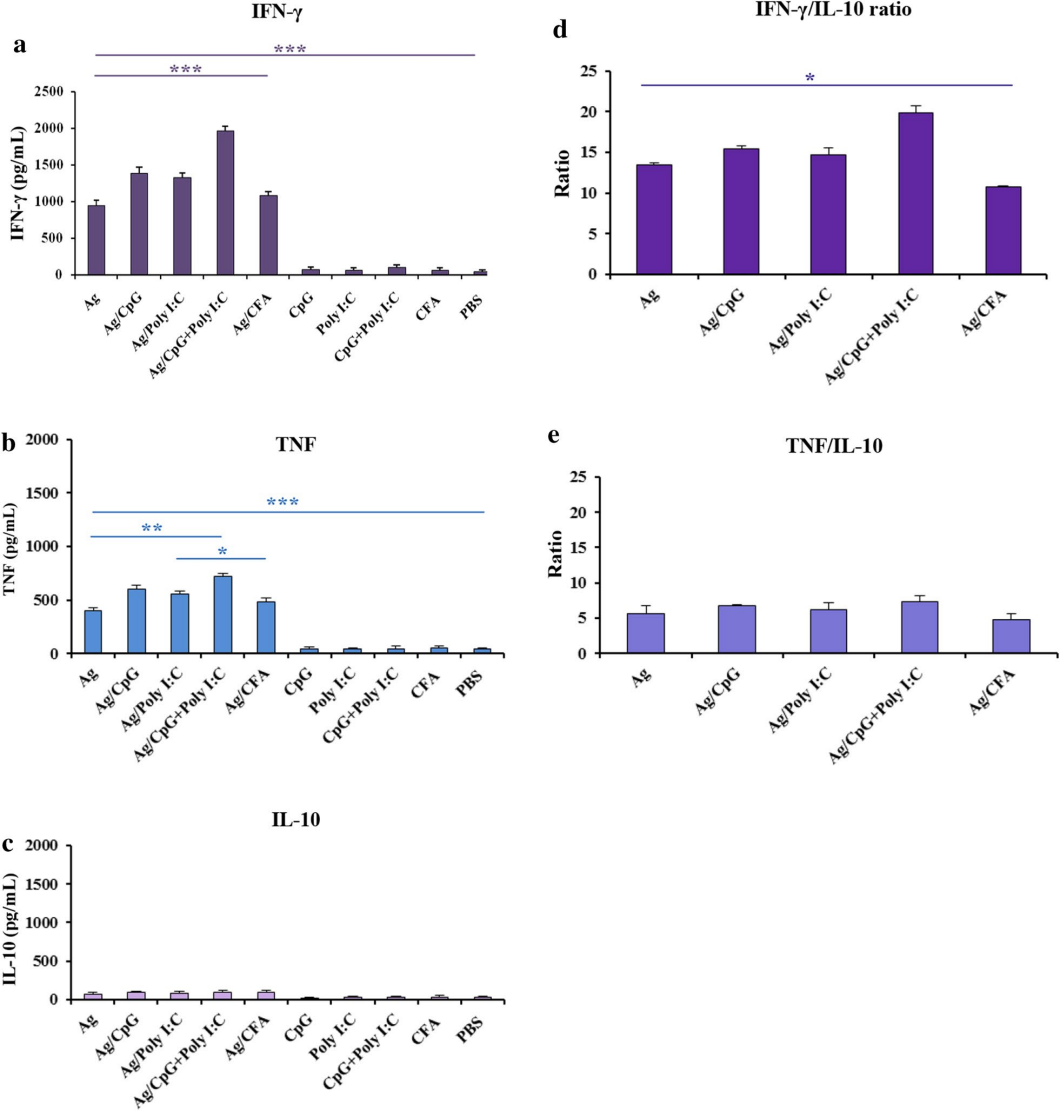
Assessment of the IFN-γ (**a**), TNF (**b**), IL-10 (**c**) production and IFN-γ/IL-10 (**d**), and TNF/IL-10 (**e**) ratios in vaccine (1–5) and control (6–10) groups. For immunized mice receiving rPfCelTOS with different adjuvants alone or in combination, the mean of IFN-γ (**a**) responses of ConA (as the positive control) and no antigen (as the negative control) were in the range of 1716–2228 and 18–27 pg/mL, respectively. The highest and significant level of IFN-γ (1959 pg/mL) was produced by the vaccine group 4 that was immunized with rPfCelTOS in combination with CpG + Poly I:C adjuvants (*P *< 0.0001, Tukey’s HSD post hoc test). Among different vaccine groups, TNF (**b**) response of ConA (as the positive control) and no antigens (as the negative control) were in the range of 981–1123 and 17–30 pg/mL, respectively. A significant difference was also observed in the level of TNF between mouse group 4 (718 pg/mL) that received rPfCelTOS/CpG + Poly I:C and mouse groups 1, 3, and 5 (rPfCelTOS, rPfCelTOS/Poly I:C and rPfCelTOS/CFA/IFA, respectively,* P* < 0.05, Tukey’s HSD post hoc test). Among different examined groups, IL-10 (**c**) responses of ConA (as the positive control) and no antigen (as the negative control) were in the range of 135–478 and 12–17 pg/mL, respectively. Analysis of IFN-γ/IL-10 (**d**) and TNF/IL-10 (**e**) ratios in the vaccine group 4 receiving rPfCelTOS/CpG + Poly I:C showed the highest ratio; however, group 5 (rPfCelTOS/CFA) presented the lowest ratios. The bars show the mean concentration of elicited IFN-γ, TNF, and IL-10 from pooled lymphocytes of immunized mice (n = 4) in each group. Data was analysed using one-way ANOVA, followed by Tukey’s HSD post hoc test. *P < 0.05, **P < 0.001, ***P < 0.0001. *Ag* rPfCelTOS antigen

Lymphocyte proliferation and cytokine production in the cultured splenocyte cells of all the vaccine groups (1–5) were determined in vitro. On day 38 of the first immunization, 4 mice from each vaccine group were anesthetized, followed by cervical dislocation, and the splenocytes were used for analysis. A significant lymphocyte proliferation in the presence of rPfCelTOS antigen was found in the vaccine groups 1–5 (mean SI: 1.95–4.33) compared with the control mouse groups (mean SI: 0.98–1.01, *P *< 0.001, one-way ANOVA; Additional file 1: Figure S4). Multiple comparisons of the proliferation level among the adjuvanted vaccine mouse group 4 (mean SI: 4.33) with groups 1–3 and group 5 showed a significant difference (*P *< 0.001, one-way ANOVA; Additional file 1: Figure S4). Supernatants of the cultured spleen cells from the vaccine groups were analysed for the presence of the cytokines IFN-γ, TNF, IL-10, and IL-4 using mouse cytokine ELISA kits (R&D System). Comparing the level of IFN-γ between the vaccine groups 1–5 and the immunized mouse control groups (6–10) showed a significant difference (*P *< 0.0001, Tukey’s HSD post hoc test; Fig. 4a). In addition, comparing the non-adjuvanted (1) and adjuvanted (2–5) vaccine groups showed that the lowest level of IFN-γ (mean: 943.4 pg/mL) was produced by the cultured spleen cells from the non-adjuvanted immunized mouse group 1 that was significantly different from adjuvanted vaccine groups 2–4 (*P *< 0.05, Tukey’s HSD post hoc test; Fig. 4a and Additional file 2: Table S2). However, in the adjuvanted vaccine groups (2–5), a statistically significant difference was found in the levels of IFN-γ in the mouse group 4 immunized with rPfCelTOS antigen formulated in CpG + Poly I:C (mean: ~ 2000 pg/mL; *P *< 0.0001, one-way ANOVA; Fig. 4a and Additional file 2: Table S2). Also, in the multiple comparisons of the vaccine groups (1–5), there was no significant difference in the level of IFN-γ between mouse group 1 (rPfCelTOS alone) and adjuvanted group 5 (Ag/CFA/IFA, as the reference adjuvant) (*P *> 0.05, Tukey’s HSD post hoc test; Fig. 4a and Additional file 2: Table S2). In adjuvanted vaccine groups (2–5), no significant difference was detected in the level of IFN-γ of group 2 (Ag/CpG) from group 3 (Ag/Poly I:C) and group 3 (Ag/Poly I:C) from group 5 (Ag/CFA/IFA) (*P *> 0.05, Tukey’s HSD post hoc test; Fig. 4a and Additional file 2: Table S2).

Analysis of TNF production in the vaccine groups immunized with rPfCelTOS in various adjuvant formulations revealed that the level of TNF in the vaccine groups 1–5 was significantly higher than the control mouse groups (*P *< 0.0001, Tukey’s HSD post hoc test; Fig. 4b). The lowest level of TNF production (mean concentration: 400.65 pg/mL) was observed in the mouse group 1 receiving rPfCelTOS alone, which was significantly different from mouse groups 2 (rPfCelTOS/CpG) and 4 (rPfCelTOS CpG + Poly I:C) (*P *< 0.05, Tukey’s HSD post hoc test; Fig. 4b and Additional file 2: Table S2). The highest level of TNF (mean concentration: ~ 720 pg/mL) was elicited in the vaccine group 4 immunized with rPfCelTOS/CpG + Poly I:C, which was significantly higher in comparison with the mouse groups 1, 3, and 5 (*P *< 0.05, Tukey’s HSD post hoc test; Fig. 4b and Additional file 2: Table S2).

Concerning the IL-4 secretion as the Th2 response cytokine, no difference in IL-4 production (< 50 pg/mL) was observed between cultured spleen cells from different immunized mice (1–5) and the control groups (6–10) that were collected either at 24 or 48 h (*P *> 0.05, one-way ANOVA). In addition, there was no statistically significant difference in the mean IL-10 concentration between the groups 1–5 (70–100 pg/mL) and the control groups 6–10 (26.5–34.7 pg/mL; *P *> 0.05, one-way ANOVA; Fig. 4c and Additional file 2: Table S2).

Analysis of IFN-γ/IL-10 ratio in the vaccine group 4 receiving rPfCelTOS in combination with two adjuvants (CpG + Poly I:C) showed a significant difference and higher ratio (19.9) than the vaccine groups 1–3 and 5 that received antigen alone or in each adjuvant individually (*P *< 0.05, Tukey’s HSD post hoc test; Fig. 4d and Additional file 2: Table S2). The lowest level of IFN-γ/IL-10 ratio was detected in mouse group 5 (10.8), which received rPfCelTOS in CFA/IFA adjuvant. The highest and lowest levels of TNF/IL-10 ratio were found in vaccine groups 4 (rPfCelTOS/CpG + Poly I:C; 7.28) and 5 (rPfCelTOS/CFA; 4.79), respectively. Multiple comparison analyses showed no statistically significant difference in TNF/IL-10 ratio in mouse groups 1–5 (*P *> 0.05, one-way ANOVA; Fig. 4e and Additional file 2: Table S2).

### Inhibition of *P. falciparum* NF54 infection in *An. stephensi* by anti-rPfCelTOS antibodies

**Fig. 5:**
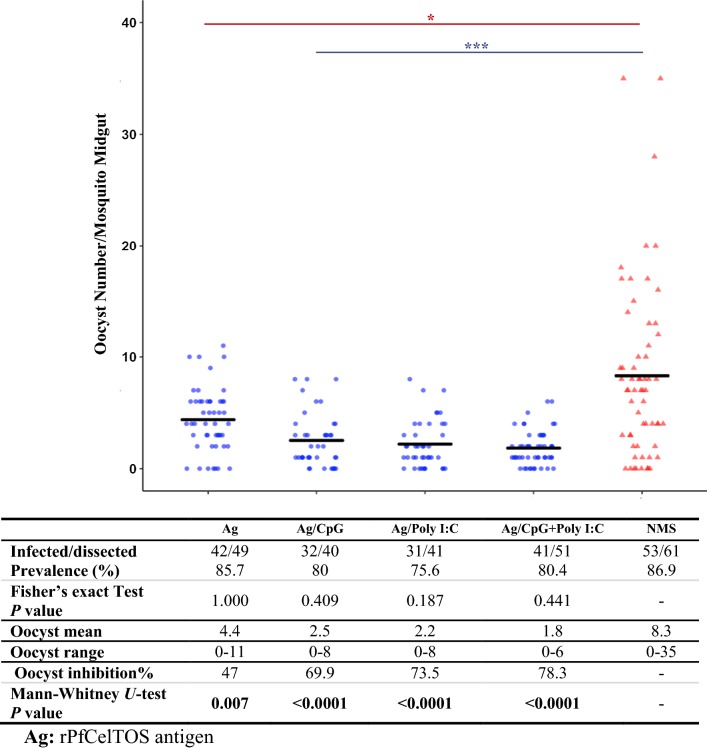
Effect of anti-rPfCelTOS antibodies on *P. falciparum* NF54 parasite infectivity in *An. stephensi* mosquitoes. Standard membrane feeding assays (SMFA) were performed on day 38 with pooled mouse sera (n = 16) of different vaccine groups (1–4) that was mixed with mature *P. falciparum* NF54 cultured gametocytes and fed to *An. stephensi* (n= 50 per cup) in SMFA. Midguts were dissected 9–10 days post feeding. A pooled NMS (n = 20) was used as the negative control. Oocyst counts revealed the successful development of *P. falciparum* in the *An. stephensi*. Two separate membrane feeds were done using serum from each vaccine group (1–4), and oocyst counts were pooled for statistical analysis. The Table shows the evaluation of *P. falciparum* infectivity and oocyst counts in different vaccine groups (1–4) and the control group. Statistical analysis (Mann–Whitney *U*-test and Fisher’s exact test) was carried out using IBM SPSS 21.0 for Windows. Data points represent the number of oocysts in individual mosquitoes, and the lines show the arithmetic mean of oocyst count. The Table shows the prevalence of infected mosquitoes, oocysts mean number, oocyst range, and percentage inhibition of oocyst in vaccine groups (1–4) relative to the control group. The Mann–Whitney *U*-test and Fisher’s exact test were used to test differences in oocyst inhibition and prevalence, respectively, between the vaccine groups and control group. *P < 0.05, **P < 0.001, ***P < 0.0001. *Ag* rPfCelTOS antigen, *NMS* normal mouse sera

To examine whether different vaccine-induced antibodies against rPfCelTOS (groups 1–4) differentially inhibit *P. falciparum* NF54 infection in *An. stephensi,* pooled mouse anti-rPfCelTOS antibodies were combined with *P. falciparum* NF54 gametocytes, and *An. stephensi* mosquitoes were permitted directly to feed. The pooled sera of vaccine groups 1–4 (rPfCelTOS, rPfCelTOS/CpG, rPfCelTOS/Poly I:C, and rPfCelTOS/CpG + Poly I:C, respectively) and pooled sera of control groups (NMS as non-adjuvant control and CpG, Poly I:C adjuvant alone or CpG + Poly I:C in combination as adjuvanted control groups) were used in SMFA, and antibodies against rPfCelTOS showed different levels of reduction in oocyst intensity of *P. falciparum* infection in *An. stephensi* (Fig. 5). Mean oocyst numbers per midgut in all different control groups ranged between 7.9 and 8.3 with no significant difference (*P *> 0.05 Mann–Whitney *U*-test; Additional file 2: Table S3). The pooled anti-rPfCelTOS antibodies from all the vaccine groups (1–4) significantly inhibited oocyst formation (47–78.3%) in *An. stephensi* relative to the NMS control group (*P *< 0.05, Mann–Whitney *U*-test; Fig. 5). The mean oocysts/midgut intensity was significantly reduced from 8.3 in the NMS control group to 1.8–2.5 in the adjuvanted vaccine groups (*P *< 0.0001 Mann–Whitney *U*-test; Fig. 5). Also, the anti-rPfCelTOS from the mouse group 4 (rPfCelTOS/CpG + Poly I:C) showed the lowest arithmetic mean oocysts (1–8) and the highest oocyst inhibition (78.3%), as compared to the vaccine groups 1–3 (Fig. 5). Furthermore, a significant difference was detected in the oocyst inhibition between the non-adjuvanted vaccine group 1 and adjuvanted vaccine groups 2–4 (*P *< 0.05, Mann–Whitney *U*-test; Table 2). Interestingly, comparison of oocyst inhibition by anti-rPfCelTOS within the adjuvanted vaccine groups 2–4 showed no significant difference (*P *> 0.05, Mann–Whitney *U*-test; Fig. 5 and Table 2). There was no significant difference in the infection prevalence of *An. stephensi* among different vaccine groups (1–4, ranging 75.6–85.7) compared with the control group (86.9) (*P *> 0.05, Fisher’s exact test; Fig. 5). The highest and the lowest infection prevalence was observed in the mouse group 1 receiving rPfCelTOS alone (42 of 49 mosquitoes examined, 85.7%) and group 3 that received antigen in Poly I:C adjuvant alone (31 of 41 mosquitoes examined, (75.6%), respectivelyv (*P *> 0.05, Fisher’s exact test; Fig. 5).

**Table 2:** Comparison of oocyst intensity between different immunization groups (1–4) by Mann–Whitney *U*-test

Compared mouse groups	Ag	Ag/CpG	Ag/Poly I:C	Ag/CpG + Poly I:C
rPfCelTOS (Ag)	–	0.002*	< 0.0001***	< 0.0001***
Ag/CpG	–	–	0.604	0.35
Ag/Poly I:C	–	–	–	0.733
Ag/CpG + Poly I:C	–	–	–	–

*Ag* rPfCelTOS antigen

*P *< 0.05 was considered statistically as significant and is shown with star(s) and italic

## Discussion

Whole parasite based malaria vaccine was the first successful vaccine trial that was achieved by irradiated sporozoites in humans in the 1950s, and the results showed protection against both homologous and heterologous parasite challenge [45]. However, in the global malaria eradication program, further large-scale production of such vaccine was challenging. Therefore, the vaccine approach was moved to “subunit malaria vaccine” that surely needs highly immunogenic parasite antigens [46]. The subunit malaria vaccines, RTS, S, which has been demonstrated to induce partial protection against malaria in phase III human clinical trials [47, 48] is supposed to become the first licensed malaria vaccine in very near future. Despite encouraging results, the level of protection is generally considered inadequate to attain the elimination of malaria [49]. Therefore, there is a great interest in improving available subunit malaria vaccines or developing new efficacious malaria subunit vaccines with better formulations to improve the immunogenicity of the target antigen that could significantly reduce disease transmission. In this concern, PfCelTOS, an advanced VIMT candidate, has a key role in the development of infections in both mosquito and human hosts [16]. Therefore, in the present work, a comparative study was performed in the immunogenicity and functional activity of rPfCelTOS delivered in Th1-based adjuvants (CpG and Poly I:C) in BALB/c mouse; the results demonstrated the ability of these vaccine formulations to elicit high-avidity antibodies that inhibit *P. falciparum* infection in *An. stephensi.*

Although early indications in preclinical animal studies of the PfCelTOS as vaccine candidate antigen are encouraging [15, 50, 51], the effort on the improvement of the vaccine formulations for efficacy induced by a PfCelTOS-based vaccine in human are under active development [52]. In this concern, it is possible that the achievement of effective PfCelTOS as a TBV may be affected by the poorly immunogenic subunit antigen. Therefore, including potent adjuvants in antigen-specific formulations might induce a robust immune response of the proper type to prevent developing parasites in both human and mosquito hosts [53]. The present study has again recommended that *E. coli*-expressed PfCelTOS is immunogenic in BALB/c mouse; however, in comparison to rPfCelTOS alone, administration of rPfCelTOS with two distinct TLR-based adjuvants (CpG and/or Poly I:C) showed an increase in responses (level of antibody, avidity, and Th1 cytokines). Also, the pattern of immunofluorescence on *P. falciparum* sporozoites (Additional file 1: Figure S2) using sera from vaccine groups 1–5 indicated CelTOS specificity.

In preclinical evaluation of candidate vaccine, it is required to consider the qualitative features of the antibody responses during the interpretation of the results such as the IgG isotypes, avidity, and functional activity. In this work, the anti-rPfCelTOS IgG subclass responses in different vaccine mouse groups were evaluated, and in non-adjuvanted group 1 that received antigen without any adjuvant, almost comparable IgG1 (as the indicator of Th2 and humoral response) and IgG2a as well as IgG2b (as the indicator of Th1 and cellular response) were detected, confirming the induction of both humoral and cellular immune responses in this vaccine group. However, in adjuvanted groups 2–4, the IgG2a subclass corresponded to a Th1 response was predominantly observed. IgG2a and IgG2b isotypes in mouse (IgG1 and IgG3 in human, respectively) are the most cytophilic and effective isotypes aiming at mediating phagocyte activation and complement fixation as in the previous studies against blood-stage antigens; these antibodies showed protection in clinical malaria in the field [54–56]. Moreover, similar to previously published work, it was reported that naturally acquired anti-rPfCelTOS response in human subjects revealed the skewed results towards cytophilic IgG1 and IgG3 [40], indicating that the presence of such isotypes may possibly explain the mechanism by which sporozoites/ookinete may be impaired.

Interestingly, among adjuvanted vaccine groups (2–4), a higher IgG2a and IgG2b responses to rPfCelTOS were observed in the adjuvanted vaccine group 4 receiving antigen with adjuvants, CpG + Poly I:C in combination. Regarding the Th-polarization responses in mouse immunized with rPfCelTOS and according to the value of IgG2a/IgG1, IgG2b/IgG1, and IgG2a + IgG2b/IgG1 + IgG3 ratios, it has been clear that rPfCelTOS alone (group 1) induces mixed Th1/Th2-responses. However, in the adjuvanted groups (2–4) that received the rPfCelTOS-based candidate vaccines formulated with CpG-ODNs, Poly I:C, and/or a combination of CpG + Poly I:C adjuvants, more shifted responses toward the Th1 immunity (cellular immunity) were detected.

In addition, the quality of pathogen-specific antibodies may play a critical role in protection following vaccination against a given pathogen [57]. In this regard, avidity that is determined as the antigen-binding ability [58] would be a good indicator, as previous works reported that the anti-CS antibody avidity with the appropriate isotype had a crucial role in mediating protection against malaria in animal model [59]. Although Th1 potent adjuvants, such as CpG and Poly I:C, increased the high avidity anti-rPfCelTOS-specific cytophilic antibodies (IgG2a and IgG2b), the combination of these two Th1 potent adjuvants (by means of different mechanisms) with recombinant protein revealed a better impact on the magnitude, high avidity, and polarization of responses toward Th1. Besides, comparing the immune responses induced by rPfCelTOS formulated in human-use compatible adjuvants (CpG and Poly I:C) with the CFA/IFA as the reference adjuvant that is inappropriate for humans use [60, 61] revealed higher and improved responses, indicating that these TLR-based adjuvants are capable of enhancement and improvement of the immunogenicity of rPfCelTOS protein.

Distinct cytokines such as IFN-*γ,* TNF, and IL-10 play an essential role in T-cell polarization. In the present work, the profile of the cytokines induced by the rPfCelTOS protein was investigated in mouse, and similar to the obtained results for the humoral immune response, it was found that the immunized mouse groups stimulated a higher level of CelTOS-specific IFN-γ and TNF. In addition, the level of rPfCelTOS-specific IFN-*γ* was reasonably higher in mouse vaccinated with rPfCelTOS adjuvanted with CpG, Poly I:C, and CpG + Poly I:C compared to the group vaccinated with rPfCelTOS protein alone, confirming again the induction of the Th1-type immune responses in these mice groups (Fig. 4a). Enhancement of the levels of rPfCelTOS-specific IFN-γ and TNF response (but marginal IL-4 response) parallel with the serological data may support their anti-parasitic role, as shown earlier [62]. It has been suggested that IL-10 has a critical role in switching from Th1 to Th2 responses. Thus, the low level of IL-10 production as well as the high ratios of IFN-*γ*/IL-10 and TNF/IL-10 in vaccine groups confirmed again switching to Th1, and this response is probably involved in controlling the infection as demonstrated before [63, 64].

Previous studies have revealed that CelTOS, as a pre-erythrocytic malaria vaccine candidate, is able to stimulate strong antibody and T cell responses, which inhibit the development of blood-stage infection in mice [13, 14, 16]. CelTOS is also expressed on ookinete in mosquito host and has an essential role in ookinete to oocyst transformation that, in fact, represents one of the most severe bottlenecks for the parasite development in mosquito host [13, 17]. Therefore, to block infection in mosquito host, anti-CelTOS antibodies should have high avidity (ability to bind), specificity, and functional activity. However, subunit protein vaccines may possibly fail to induce strong and sterile immunity, as observed with other TBV subunit candidate, Pfs25 [65]. Hence, this limitation suggests the significance of testing subunit antigen with proper, potent and strong adjuvants. Therefore, one of the most important qualitative aspects of the antibody responses to rPfCelTOS antigen evaluated in the present investigation was the assessment of anti-rPfCelTOS antibodies to inhibit oocysts development in female *An. stephensi* using SMFA. In the present work, IgG raised against *E. coli*-expressed PfCelTOS protein from vaccine groups 1–4 demonstrated functional activity on parasite infectivity using *P. falciparum* NF54 parasite in *An. stephensi*, thereby again validating it as a potent candidate for the malaria TBV subunit. However, among vaccine groups (1–4), antibodies from the adjuvanted groups (2–4) showed an enhancement in oocyst inhibitory effect. More specifically, among the adjuvanted groups, the strongest inhibitory antibodies were from group 4, indicating that co-adminstration of two distinct TLRs (3 and 9) vaccine adjuvants with rPfCelTOS antigen induces better anti-rPfCelTOS-specific antibodies in avidity and functional effects due to generating multiple signalings. In fact, such polyclonal antibodies could bind strongly to CelTOS protein on ookinetes and to inhibit/block further oocysts development in the basal lamina of mosquito host. However, on the contrary to the obtained results with these polyclonal antibodies in the present study, Espinosa et al. [17] have reported the inhibitory effect of monoclonal antibodies (MAb3C3 and MAb 4D10), but not mice polyclonal sera, against PfCelTOS through *P. falciparum* oocyst formation in Anopheles mosquitoes. Therefore, these polyclonal antibodies without further IgG purification showed ability to inhibit oocysts development in mosquito host, supporting the protective effect of anti-rPfCelTOS responses that has been reported previously [13–15, 17, 66] and provide additional evidence of its key role as a target for VIMT development.

## Conclusions

Taken together, a key finding in this investigation is that BALB/c mouse immunization with the CelTOS protein leads to the activation of both humoral and cellular immune responses and induces anti-rPfCelTOS antibodies that were reasonably able to bind and recognize native CelTOS expressed by the *P. falciparum* sporozoites. Immunological characteristics (IgG level, cytophilic IgG2a and IgG2b, avidity, and Th1 cytokines) and TRA of anti-rPfCelTOS significantly enhanced in the presence of co-administration of TLR-3 and -9 as human-use compatible adjuvants, confirming that targeting TLRs would be an effective route for enhancing the induction of TRA against rPfCelTOS. Such antibodies could bind to CelTOS on ookinetes and to inhibit/block their traversal through the mosquito midgut epithelial cells, thus abolishing development to oocysts in the basal lamina. Therefore, targeting both sporozoites and ookinetes by anti-rPfCelTOS could be more effective due the induction of immune responses that obstruct parasite development at multiple stages in both human and mosquito hosts.

## Additional files

**Additional file 1: Figure S1.** Western blot analysis of expressed PfCelTOS in *E. coli*. **Figure S2.** Indirect immunofluorescence antibody test (IFAT). **Figure S3.** Comparison of the anti-rPfCelTOS IgG, IgG1, IgG2a, IgG2b, and IgG3 antibodies in all vaccinated mice groups (1–5) with the control mice groups (6–10) on day 38 of the first immunization. **Figure S4**. Lymphocyte proliferation in cultured splenocyte cells of immunized mice (groups 1–10) in the presence of rPfCelTOS in vitro.

**Additional file 2: Table S1.** Multiple comparisons of means anti-rPfCelTOS IgG, its subclasses, Th1/Th2 ratio and anti-rPfCelTOS avidity antibodies among the non-adjuvanted (group 1) and adjuvanted (groups 2—5) vaccine groups on day 38 of the first immunization using Tukey’s HSD post hoc test. **Table S2**. Multiple comparisons of mean IFN-γ, TNF, and IL-10 cytokines levels, IFN-γ/IL-10 and TNF/IL-10 ratios, and stimulation Index (SI) of MTT assay among all vaccine groups (1—5) with Tukey’s HSD post hoc test. **Table S3.** Effect of anti-rPfCelTOS IgG antibodies induced in mice on *P. falciparum* infectivity in *An. stephensi.*

## Abbreviations

AI: avidity index
CFA: complete Freund’s adjuvant
ConA: concanavalin A
CpG ODN: oligodeoxynucleotides composed of unmethylated cytosine phosphate guanidine motifs
dsRNA: double-stranded RNA
ELISA: enzyme-linked immunosorbent assay
FITC: fluorescein isothiocyanate
h: hour
HRP: horseradish peroxidase
HSD: Tukey’s honestly significant difference
IFA: incomplete Freund’s adjuvant
IFAT: indirect immunofluorescence antibody test
IPTG: isopropyl-beta-d-1-thiogalactopyranoside
malERA: Malaria Eradication Research Agenda
2-ME: 2-mercaptoethanol
MTT: 3-(4,5-dimethylthiazol-2-yl)-2,5-diphenyltetrazolium bromide
PBS: phosphate-buffered saline
PBS-T: PBS 1× containing 0.05% Tween 20
PfCelTOS: *P. falciparum* cell-traversal protein for ookinetes and sporozoites
Poly I: C: polyinosinic:polycytidylic acid
RT: room temperature
SD: standard deviation
SI: stimulation index
SDS: sodium dodecyl sulfate
SDS-PAGE: SDS–polyacrylamide gel electrophoresis
SMFA: standard membrane feeding assay
TB: terrific broth
TBV: transmission-blocking vaccine
TLR: toll-like receptor
TRA: transmission-reducing activity
VIMT: vaccine that interrupts malaria transmission

## References

[1] WHO. Fact sheets malaria. Geneva, World Health Organization, 2018. http://www.who.int/news-room/fact-sheets/detail/malaria. Accessed 19 November 2018.

[2] World Health Assembly, 33. Declaration of global eradication of smallpox. World Health Organization. 1980. http://www.who.int/iris/handle/10665/155528. Accessed 1980.

[3] Bhattacharya S (2008). The World Health Organization and global smallpox eradication. J Epidemiol Community Health.

[4] de Quadros CA, Andrus JK, Olive JM, Guerra de Macedo C, Henderson DA (1992). Polio eradication from the Western Hemisphere. Annu Rev Public Health.

[5] Alonso PL, Brown G, Arevalo-Herrera M, Binka F, Chitnis C, Collins F (2011). A research agenda to underpin malaria eradication. PLos Med..

[6] malERA Consultative Group on Vaccines (2011). A research agenda for malaria eradication: vaccines. PLoS Med..

[7] The malERA Consultative Panel on Tools for Malaria Elimination (2017). malERA: an updated research agenda for diagnostics, drugs, vaccines, and vector control in malaria elimination and eradication. PLoS Med..

[8] Carter R, Mendis KN, Miller LH, Molineaux L, Saul A (2000). Malaria transmission-blocking vaccines how can their development be supported?. Nat Med.

[9] Riley EM, Stewart VA (2013). Immune mechanisms in malaria: new insights in vaccine development. Nat Med.

[10] Birkett AJ, Moorthy VS, Loucq C, Chitnis CE, Kaslow DC (2013). Malaria vaccine R&D in the decade of vaccines: breakthroughs, challenges and opportunities. Vaccine..

[11] Jimah JR, Salinas ND, Sala-Rabanal M, Jones NG, Sibley LD, Nichols CG (2016). Malaria parasite CelTOS targets the inner leaflet of cell membranes for pore-dependent disruption. Elife..

[12] Aguiar JC, Bolton J, Wanga J, Sacci JB, Iriko H, Mazeika JK (2015). Discovery of novel *Plasmodium falciparum* pre-erythrocytic antigens for vaccine development. PLoS ONE.

[13] Bergmann-Leitner ES, Mease RM, De La Vega P, Savranskaya T, Polhemus M, Ockenhouse C (2010). Immunization with pre-erythrocytic antigen CelTOS from *Plasmodium falciparum* elicits cross-species protection against heterologous challenge with *Plasmodium berghei*. PLoS ONE.

[14] Bergmann-Leitner ES, Legler PM, Savranskaya T, Ockenhouse CF, Angov E (2011). Cellular and humoral immune effector mechanisms required for sterile protection against sporozoite challenge induced with the novel malaria vaccine candidate CelTOS. Vaccine..

[15] Bergmann-Leitner ES, Hosie H, Trichilo J, Deriso E, Ranallo RT, Alefantis T (2013). Self-adjuvanting bacterial vectors expressing pre-erythrocytic antigens induce sterile protection against malaria. Front Immunol..

[16] Kariu T, Ishino T, Yano K, Chinzei Y, Yuda M (2006). CelTOS, a novel malarial protein that mediates transmission to mosquito and vertebrate hosts. Mol Microbiol.

[17] Espinosa DA, Vega-Rodriguez J, Flores-Garcia Y, Noe AR, Muñoz C, Coleman R (2017). The *Plasmodium falciparum* cell-traversal protein for ookinetes and sporozoites as a candidate for preerythrocytic and transmission-blocking vaccines. Infect Immun.

[18] Mbow ML, De Gregorio E, Valiante NM, Rappuoli R (2010). New adjuvants for human vaccines. Curr Opin Immunol.

[19] Bevan MJ (2004). Helping the CD8(+) T-cell response. Nat Rev Immunol.

[20] Dasari P, Nicholson IC, Hodge G, Dandie GW, Zola H (2005). Expression of toll-like receptors on B lymphocytes. Cell Immunol.

[21] O’Neill LA, Bryant CE, Doyle SL (2009). Therapeutic targeting of toll-like receptors for infectious and inflammatory diseases and cancer. Pharmacol Rev.

[22] Harandi AM, Holmgren J (2004). CpG DNA as a potent inducer of mucosal immunity: implications for immunoprophylaxis and immunotherapy of mucosal infections. Curr Opin Investig Drugs.

[23] Halperin SA, Dobson S, McNeil S, Langley JM, Smith B, McCall-Sani R (2006). Comparison of the safety and immunogenicity of hepatitis B virus surface antigen co-administered with an immunostimulatory phosphorothioate oligonucleotide and a licensed hepatitis B vaccine in healthy young adults. Vaccine..

[24] Barry M, Cooper C (2007). Review of hepatitis B surface antigen-1018 ISS adjuvant-containing vaccine safety and efficacy. Expert Opin Biol Ther..

[25] Gupta GK, Agrawal DK (2010). CpG oligodeoxynucleotides as TLR9 agonists: therapeutic application in allergy and asthma. Bio Drugs..

[26] Schmidt C (2007). Clinical setbacks for toll-like receptor 9 agonists in cancer. Nat Biotechnol.

[27] Krieg AM (2004). Anti tumor applications of stimulating Toll-like receptor 9 with CpG oligodeoxynucleotides. Curr Oncol Rep..

[28] Alexopoulou L, Holt AC, Medzhitov R, Flavell RA (2001). Recognition of double-stranded RNA and activation of NF-κB by Toll-like receptor 3. Nature.

[29] Duthie MS, Windish HP, Fox CB, Reed SG (2011). Use of defined TLR ligands as adjuvants within human vaccines. Immunol Rev.

[30] Schulz O, Diebold SS, Chen M, Näslund TI, Nolte MA, Alexopoulou L (2005). Toll-like receptor 3 promotes cross-priming to virus-infected cells. Nature.

[31] Longhi MP, Trumpfheller C, Idoyaga J, Caskey M, Matos I, Kluger C (2009). Dendritic cells require a systemic type I interferon response to mature and induce CD4+ Th1 immunity with poly IC as adjuvant. J Exp Med.

[32] Davey GM, Wojtasiak M, Proietto AI, Carbone FR, Heath WR, Bedoui S (2010). Cutting edge: priming of CD8 T cell immunity to herpes simplex virus type 1 requires cognate TLR3 expression in vivo. J Immunol..

[33] Trumpfheller C, Caskey M, Nchinda G, Longhi MP, Mizenina O, Huang Y (2008). The microbial mimic poly IC induces durable and protective CD4+ T cell immunity together with a dendritic cell targeted vaccine. Proc Natl Acad Sci USA.

[34] Apostólico S, Boscardin SB, Yamamoto MM, Oliveira-Filho JN, Kalil J, Cunha-Neto E (2016). HIV envelope trimer specific immune response is influenced by different adjuvant formulations and heterologous prime-boost. PLoS ONE..

[35] Henriques HR, Rampazo EV, Gonçalves AJ, Vicentin EC, Amorim JH, Panatieri RH (2013). Targeting the non-structural protein 1 from dengue virus to a dendritic cell population confers protective immunity to lethal virus challenge. PLoS Negl Trop Dis..

[36] Tewari K, Flynn BJ, Boscardin SB, Kastenmueller K, Salazar AM, Anderson CA (2010). Poly(I:C) is an effective adjuvant for antibody and multi-functional CD4+ T cell responses to *Plasmodium falciparum* circumsporozoite protein (CSP) and αDEC-CSP in non human primates. Vaccine..

[37] Forte G, Rega A, Morello S, Luciano A, Arra C, Pinto A (2012). Polyinosinic-polycytidylic acid limits tumor outgrowth in a mouse model of metastatic lung cancer. J Immunol..

[38] Nagato T, Lee YR, Harabuchi Y, Celis E (2014). Combinatorial immunotherapy of polyinosinic-polycytidylic acid and blockade of programmed death-ligand 1 induce effective CD8 T-cell responses against established tumors. Clin Cancer Res.

[39] Moorthy VS, Newman RD, Okwo-Bele JM (2013). Malaria vaccine technology roadmap. Lancet.

[40] Pirahmadi S, Zakeri S, Mehrizi AA, Karimi L, Djadid ND (2019). Heterogeneity in the acquisition of naturally acquired antibodies to cell-traversal protein for ookinetes and sporozoites (CelTOS) and thrombospondin-related adhesion protein (TRAP) of *Plasmodium falciparum* in naturally infected patients from seasonal and unstable malaria in Iran. Acta Trop.

[41] Mehrizi AA, Zakeri S, Rafati S, Salmanian AH, Djadid ND (2011). Immune responses elicited by co-immunization of *Plasmodium vivax* and *P. falciparum* MSP-1 using prime-boost immunization strategies. Parasite Immunol..

[42] Hedman K, Lappalainen M, Seppaia I, Makela O (1989). Recent primary toxoplasma infection indicated by a low avidity of specific IgG. J Infect Dis.

[43] Fivelman QL, McRobert L, Sharp S, Taylor CJ, Saeed M, Swales CA (2007). Improved synchronous production of *Plasmodium falciparum* gametocytes *in vitro*. Mol Biochem Parasitol.

[44] Carter R, Miller LH (1979). Evidence for environmental modulation of gametocytogenesis in *Plasmodium falciparum* in continuous culture. Bull World Health Organ.

[45] Hoffman SL, Goh LM, Luke TC, Schneider I, Le TP, Doolan DL (2002). Protection of humans against malaria by immunization with radiation-attenuated *Plasmodium falciparum* sporozoites. J Infect Dis.

[46] Nussenzweig V, Nussenzweig RS (1985). Circumsporozoite proteins of malaria parasites. Cell.

[47] RTSS Clinical Trials Partnership (2015). Efficacy and safety of RTS, S/AS01 malaria vaccine with or without a booster dose in infants and children in Africa: final results of a phase 3, individually randomised, controlled trial. Lancet.

[48] Casares S, Brumeanu TD, Richie TL (2010). The RTS, S malaria vaccine. Vaccine..

[49] Arama C, Troye-Blomberg M (2014). The path of malaria vaccine development: challenges and perspectives. J Intern Med.

[50] Fox CB, Baldwin SL, Vedvick TS, Angov E, Reed SG (2012). Effects on immunogenicity by formulations of emulsion-based adjuvants for malaria vaccines. Clin Vacc Immunol..

[51] Voepel N, Boes A, Edgue G, Beiss V, Kapelski S, Reimann A (2014). Malaria vaccine candidate antigen targeting the pre-erythrocytic stage of *Plasmodium falciparum* produced at high level in plants. Biotechnol J.

[52] Draper SJ, Angov E, Horii T, Miller LH, Srinivasan P, Theisen M (2015). Recent advances in recombinant protein-based malaria vaccines. Vaccine..

[53] Schijns VE, Lavelle EC (2011). Trends in vaccine adjuvants. Exp Rev Vacc.

[54] Rzepczyk CM, Hale K, Woodroffe N, Bobogare A, Csurhes P, Ishii A (1997). Humoral immune responses of Solomon Islanders to the merozoite surface antigen 2 of *Plasmodium falciparum* show pronounced skewing towards antibodies of the immunoglobulin G3 subclass. Infect Immun.

[55] Taylor RR, Allen SJ, Greenwood BM, Riley EM (1998). IgG3 antibodies to *Plasmodium falciparum* merozoite surface protein 2 (MSP2): increasing prevalence with age and association with clinical immunity to malaria. Am J Trop Med Hyg.

[56] Daher LJ, Demanga CG, Prieur E, Pérignon JL, Bouharoun-Tayoun H, Druilhe P (2010). Toward the rational design of a malaria vaccine construct using the MSP3 family as an example: contribution of immunogenicity studies in models. Infect Immun.

[57] Lambert PH, Liu M, Siegrist CA (2005). Can successful vaccines teach us how to induce efficient protective immune responses?. Nat Med.

[58] Goldblatt D, Vaz AR, Miller E (1998). Antibody avidity as a surrogate marker of successful priming by *Haemophilus influenzae* type b conjugate vaccines following infant immunization. J Infect Dis.

[59] Reed RC, Louis-Wileman V, Wells RL, Verheul AF, Hunter RL, Lal AA (1996). Re-investigation of the circumsporozoite protein-based induction of sterile immunity against *Plasmodium berghei* infection. Vaccine..

[60] Hughes LE, Kearney R, Tully M (1970). A study in clinical cancer immunotherapy. Cancer.

[61] Stills HF (2005). Adjuvants and antibody production: dispelling the myths associated with Freund’s complete and other adjuvants. ILAR J.

[62] Mordmüller BG, Metzger WG, Juillard P, Brinkman BM, Verweij CL, Grau GE (1997). Tumor necrosis factor in *Plasmodium falciparum* malaria: high plasma level is associated with fever, but high production capacity is associated with rapid fever clearance. Eur Cytokine Netw.

[63] Kobayashi F, Morii T, Matsui T, Fujino T, Watanabe Y, Weidanz WP (1996). Production of interleukin 10 during malaria caused by lethal and nonlethal variants of *Plasmodium yoelii yoelii*. Parasitol Res.

[64] Yoshida A, Maruyama H, Kumagai T, Amano T, Kobayashi F, Zhang M (2000). *Schistosoma mansoni* infection cancels the susceptibility to *Plasmodium chabaudi* through induction of type 1 immune responses in A/J mice. Int Immunol.

[65] Talaat KR, Ellis RD, Hurd J, Hentrich A, Gabriel E, Hynes NA (2016). Safety and immunogenicity of Pfs25-EPA/Alhydrogel^®^, a transmission blocking vaccine against *Plasmodium falciparum*: an open label study in malaria naïve adults. PLoS ONE.

[66] Bergmann-Leitner ES, Chaudhury S, Steers NJ, Sabato M, Delvecchio V, Wallqvist AS (2013). Computational and experimental validation of B and T-cell epitopes of the in vivo immune response to a novel malarial antigen. PLoS ONE.

