# Supplementary material for: Cell-traversal protein for ookinetes and sporozoites (CelTOS) formulated with potent TLR adjuvants induces high-affinity antibodies that inhibit Plasmodium falciparum infection in Anopheles stephensi
Source: Malar J. 2019 Apr 24;18:146. doi: 10.1186/s12936-019-2773-3 (PMC6480871; doi:10.1186/s12936-019-2773-3)
Supplement: Supplementary file 1 — Additional file 1: Figure S1. Western blot analysis of expressed PfCelTOS in E. coli. Figure S2. Indirect immunofluorescence antibody test (IFAT). Figure S3. Comparison of the anti-rPfCelTOS IgG, IgG1, IgG2a, IgG2b, and IgG3 antibodies in all vaccinated mice groups (1–5) with the control mice groups (6–10) on day 38 of the first immunization. Figure S4. Lymphocyte proliferation in cultured splenocyte cells of immunized mice (groups 1–10) in the presence of rPfCelTOS in vitro. [file 12936_2019_2773_MOESM1_ESM.zip › Additional file 1 Figures/Additional file 1Figure S1.pdf]

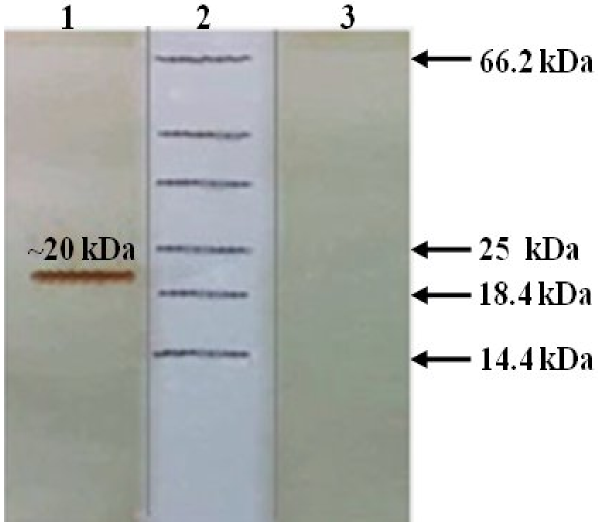

Additional file 1: Figure S1

Western blot analysis of expressed PfCelTOS in *E. coli*.

Western blot analysis of rPfCelTOS using pooled sera derived from the immunized mice with rPfCelTOS (groups 1–5) showed that anti-rPfCelTOS IgG antibodies recognized the purified rPfCelTOS antigen (a single band with a molecular mass of ~ 20 kDa, lane 1). Molecular weight marker (MW, Thermo Fisher Scientific, USA) was shown in lane 2. There was no recognition of rPfCelTOS by normal mouse sera (lane 3), indicating rPfCelTOS-specific responses.
