# Supplementary material for: Cell-traversal protein for ookinetes and sporozoites (CelTOS) formulated with potent TLR adjuvants induces high-affinity antibodies that inhibit Plasmodium falciparum infection in Anopheles stephensi
Source: Malar J. 2019 Apr 24;18:146. doi: 10.1186/s12936-019-2773-3 (PMC6480871; doi:10.1186/s12936-019-2773-3)
Supplement: Supplementary file 1 — Additional file 1: Figure S1. Western blot analysis of expressed PfCelTOS in E. coli. Figure S2. Indirect immunofluorescence antibody test (IFAT). Figure S3. Comparison of the anti-rPfCelTOS IgG, IgG1, IgG2a, IgG2b, and IgG3 antibodies in all vaccinated mice groups (1–5) with the control mice groups (6–10) on day 38 of the first immunization. Figure S4. Lymphocyte proliferation in cultured splenocyte cells of immunized mice (groups 1–10) in the presence of rPfCelTOS in vitro. [file 12936_2019_2773_MOESM1_ESM.zip › Additional file 1 Figures/Additional file 1Figure S2.pdf]

a b

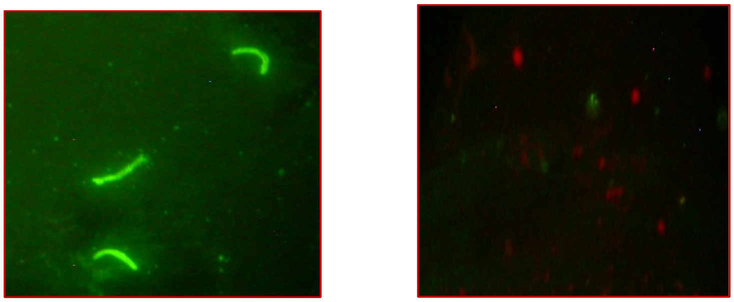

Additional file 1: Figure S2

Representative photographs of indirect immunofluorescence antibody test (IFAT). IFAT was performed to assess the ability of anti-rPfCelTOS sera of the immunized mice to recognize the native form of the CelTOS antigen on the sporozoite surface of *P. falciparum*. Multi-spot slides (a kind gift from Prof. G. Snounou), which were coated with 20 µL/well of *P. falciparum* sporozoites were blocked with 20 µL of PBS 1× containing 2.5% BSA (Roche, Basel, Switzerland) in a dark humidified chamber at room temperature for 30 min. After the slides were washed three times with PBS 1× (pH 7.4), a dilution of anti-rPfCelTOS polyclonal mouse serum in PBS 1× (1:200) was added to the duplicated wells and incubated in a wet chamber for 60 min. After three-times washing with PBS 1× (pH 7.4), each well was covered with 20 µL of the fluorescein isothiocyanate (FITC)-labeled rabbit anti-mouse IgG (1:40) and Evans blue (1:100) and then left in a wet chamber for 45 min. Each slide was examined under a fluorescence microscope (Nikon E200, Tokyo, Japan) with an oil immersion objective (100×). The serum samples obtained from the normal mouse were used as negative controls. The representative pictures show the recognition of native form of PfCelTOS in the surface of *P. falciparum* sporozoite using polyclonal antibodies (1:200) produced in mouse against rPfCelTOS. (**a**) Pooled sera derived from the immunized mice with rPfCelTOS (groups 1–5). There was no recognition

of native protein on *P. falciparum* parasite by control mouse sera (**b**), indicating rPfCelTOS-specific responses.
