# Supplementary material for: Cell-traversal protein for ookinetes and sporozoites (CelTOS) formulated with potent TLR adjuvants induces high-affinity antibodies that inhibit Plasmodium falciparum infection in Anopheles stephensi
Source: Malar J. 2019 Apr 24;18:146. doi: 10.1186/s12936-019-2773-3 (PMC6480871; doi:10.1186/s12936-019-2773-3)
Supplement: Supplementary file 1 — Additional file 1: Figure S1. Western blot analysis of expressed PfCelTOS in E. coli. Figure S2. Indirect immunofluorescence antibody test (IFAT). Figure S3. Comparison of the anti-rPfCelTOS IgG, IgG1, IgG2a, IgG2b, and IgG3 antibodies in all vaccinated mice groups (1–5) with the control mice groups (6–10) on day 38 of the first immunization. Figure S4. Lymphocyte proliferation in cultured splenocyte cells of immunized mice (groups 1–10) in the presence of rPfCelTOS in vitro. [file 12936_2019_2773_MOESM1_ESM.zip › Additional file 1 Figures/Additional file 1Figure S3.pdf]

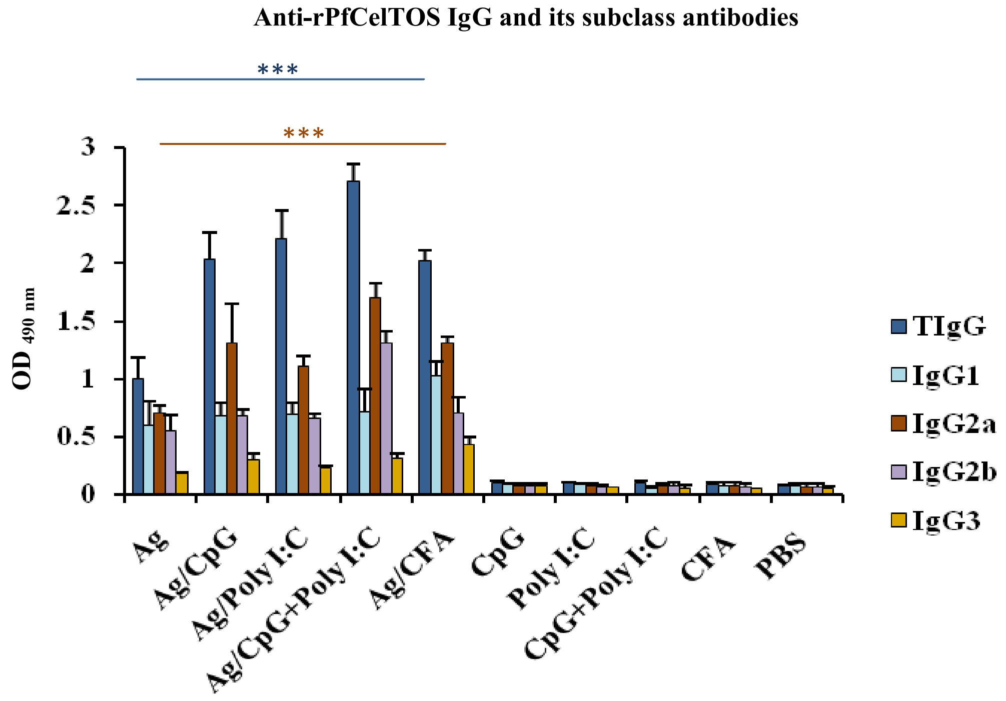

Additional file 1: Figure S3

Comparison of the anti-rPfCelTOS IgG, IgG1, IgG2a, IgG2b, and IgG3 antibodies in all vaccinated mice groups (1-5) and control mice groups (6-10) on day 38 of the first immunization. Control mice groups received PBS 1× alone or in combination with CpG, Poly I:C, CpG + Poly I:C, and CFA/IFA adjuvants. All experiments were performed with 1:200 dilution sera. The bars and error bars show the mean OD_{490nm} and standard deviation (SD) in each mouse group (n = 16), respectively. Data represents means from three separate ELISA experiments. The level of IgG and IgG2a in the non-adjuvanted mice group 1 that received rPfCelTOS alone were significantly lower than adjuvanted mice groups 2-5 that received rPfCelTOS antigen formulated in CpG, Poly I:C adjuvant alone, in combination with CpG + Poly I:C, and reference adjuvant CFA/IFA ($P < 0.0001$, one-way ANOVA).

***$P < 0.0001$

Ag rPfCelTOS, *TIgG* Total IgG
