# Supplementary material for: Cell-traversal protein for ookinetes and sporozoites (CelTOS) formulated with potent TLR adjuvants induces high-affinity antibodies that inhibit Plasmodium falciparum infection in Anopheles stephensi
Source: Malar J. 2019 Apr 24;18:146. doi: 10.1186/s12936-019-2773-3 (PMC6480871; doi:10.1186/s12936-019-2773-3)
Supplement: Supplementary file 1 — Additional file 1: Figure S1. Western blot analysis of expressed PfCelTOS in E. coli. Figure S2. Indirect immunofluorescence antibody test (IFAT). Figure S3. Comparison of the anti-rPfCelTOS IgG, IgG1, IgG2a, IgG2b, and IgG3 antibodies in all vaccinated mice groups (1–5) with the control mice groups (6–10) on day 38 of the first immunization. Figure S4. Lymphocyte proliferation in cultured splenocyte cells of immunized mice (groups 1–10) in the presence of rPfCelTOS in vitro. [file 12936_2019_2773_MOESM1_ESM.zip › Additional file 1 Figures/Additional file 1Figure S4.pdf]

Proliferation of splenocytes

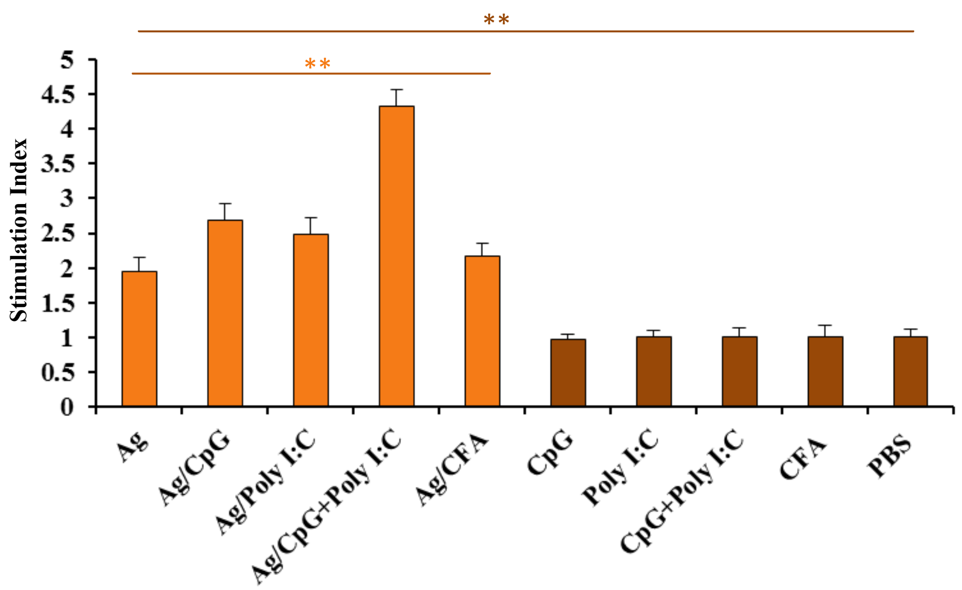

Additional file 1: Figure S4

Lymphocyte proliferation in cultured splenocyte cells of immunized mice (groups 1-10) in the presence of rPfCelTOS *in vitro*. Spleens from each vaccine (1-5) and control (6-10) groups (four mice per group) were removed, and lymphocyte proliferation was evaluated using the MTT assay. A significant lymphocyte proliferation in the presence of rPfCelTOS antigen was observed in mice group 4 in comparison with groups 1-3, and 5 ($P < 0.001$, one-way ANOVA). Data was analyzed using one way ANOVA followed by Tukey's HSD post hoc test.

**$P < 0.001$.

Ag rPfCelTOS antigen
