# Supplementary material for: Cell-traversal protein for ookinetes and sporozoites (CelTOS) formulated with potent TLR adjuvants induces high-affinity antibodies that inhibit Plasmodium falciparum infection in Anopheles stephensi
Source: Malar J. 2019 Apr 24;18:146. doi: 10.1186/s12936-019-2773-3 (PMC6480871; doi:10.1186/s12936-019-2773-3)
Supplement: Supplementary file 2 — Additional file 2: Table S1. Multiple comparisons of means anti-rPfCelTOS IgG, its subclasses, Th1/Th2 ratio and anti-rPfCelTOS avidity antibodies among the non-adjuvanted (group 1) and adjuvanted (groups 2—5) vaccine groups on day 38 of the first immunization using Tukey’s HSD post hoc test. Table S2. Multiple comparisons of mean IFN-γ, TNF, and IL-10 cytokines levels, IFN-γ/IL-10 and TNF/IL-10 ratios, and stimulation Index (SI) of MTT assay among all vaccine groups (1—5) with Tukey’s HSD post hoc test. Table S3. Effect of anti-rPfCelTOS IgG antibodies induced in mice on P. falciparum infectivity in An. stephensi. [file 12936_2019_2773_MOESM2_ESM.zip › Additional file2 Tables/Additional file 2 Table S1.pdf]

Additional file 2: Table S1 Multiple comparisons of means anti-rPfCelTOS IgG, its subclasses, Th1/Th2 ratio and anti-rPfCelTOS avidity antibodies among the non-adjuvanted (group 1) and adjuvanted (groups 2–5) vaccine groups on day 38 of the first immunization using Tukey's HSD post hoc test.

Compared Mouse Groups							Th1/Th2 ratio			Avidity		
		**TIgG**	**IgG1**	**IgG2a**	**IgG2b**	**IgG3**	**IgG2a/IgG1**	**IgG2b/IgG1**	**IgG2a+IgG2b/ IgG1+IgG3**	**TIgG**	**IgG2a**	**IgG2b**
**Ag (rPfCelTOS)**	**Ag/CpG**	**<0.0001*****	**0.045***	**<0.0001*****	**0.038***	**<0.0001*****	**<0.0001*****	0.489	**0.007***	**<0.0001*****	**<0.0001*****	**<0.0001*****
	**Ag/Poly I:C**	**<0.0001*****	0.73	**<0.0001*****	0.236	0.361	**<0.0001*****	1.000	**0.028***	**<0.0001*****	**<0.0001*****	**<0.0001*****
	**Ag/CpG+Poly I:C**	**<0.0001*****	0.502	**<0.0001*****	**<0.0001*****	**<0.0001*****	**<0.0001*****	**<0.0001*****	**<0.0001*****	**<0.0001*****	**<0.0001*****	**<0.0001*****
	**Ag/CFA/IFA**	**<0.0001*****	**<0.0001*****	**<0.0001*****	**0.012***	**<0.0001*****	0.561	**<0.0001*****	0.065	**<0.0001*****	**<0.0001*****	**<0.0001*****
**Ag/CpG**	**Ag/Poly I:C**	0.426	0.73	0.079	0.995	**0.001****	**<0.0001*****	0.741	0.5	**0.037***	**0.033***	0.221
	**Ag/CpG+Poly I:C**	**<0.0001*****	0.881	**0.001****	**<0.0001*****	0.965	**<0.0001*****	**<0.0001*****	**<0.0001*****	**<0.0001*****	**<0.0001*****	**0.002***
	**Ag/CFA/IFA**	1.000	**0.011***	0.998	1.000	**<0.0001*****	**<0.0001*****	**<0.0001*****	**0.001****	1.000	**<0.0001*****	0.267
**Ag/Poly I:C**	**Ag/CpG+Poly I:C**	**<0.0001*****	1.000	**<0.0001*****	**<0.0001*****	**<0.0001*****	**<0.0001*****	**<0.0001*****	**<0.0001*****	**0.005***	0.828	0.754
	**Ag/CFA/IFA**	0.387	**<0.0001*****	0.322	0.917	**<0.0001*****	**<0.0001*****	**<0.0001*****	**0.002***	**0.021***	**<0.0001*****	**<0.0001*****
**Ag/CpG+Poly I:C**	**Ag/CFA/IFA**	**<0.0001*****	**<0.0001*****	**<0.0001*****	**<0.0001*****	**<0.0001*****	**<0.0001*****	**<0.0001*****	**<0.0001*****	**<0.0001*****	**<0.0001*****	**<0.0001*****

$P < 0.05$ were considered statistically significant and shown with star(s) and bold (*$P < 0.05$, **$P \leq 0.001$, ***$P < 0.0001$)

TIgG total IgG
