# Supplementary material for: Cell-traversal protein for ookinetes and sporozoites (CelTOS) formulated with potent TLR adjuvants induces high-affinity antibodies that inhibit Plasmodium falciparum infection in Anopheles stephensi
Source: Malar J. 2019 Apr 24;18:146. doi: 10.1186/s12936-019-2773-3 (PMC6480871; doi:10.1186/s12936-019-2773-3)
Supplement: Supplementary file 2 — Additional file 2: Table S1. Multiple comparisons of means anti-rPfCelTOS IgG, its subclasses, Th1/Th2 ratio and anti-rPfCelTOS avidity antibodies among the non-adjuvanted (group 1) and adjuvanted (groups 2—5) vaccine groups on day 38 of the first immunization using Tukey’s HSD post hoc test. Table S2. Multiple comparisons of mean IFN-γ, TNF, and IL-10 cytokines levels, IFN-γ/IL-10 and TNF/IL-10 ratios, and stimulation Index (SI) of MTT assay among all vaccine groups (1—5) with Tukey’s HSD post hoc test. Table S3. Effect of anti-rPfCelTOS IgG antibodies induced in mice on P. falciparum infectivity in An. stephensi. [file 12936_2019_2773_MOESM2_ESM.zip › Additional file2 Tables/Additional file 2 Table S2.pdf]

Additional file 2:Table S2 Multiple comparisons of mean level of IFN-γ, TNF-α, and IL-10 cytokines, IFN-γ/IL-10 and TNF-α/IL-10 ratios and stimulation Index (SI) of MTT assay among all vaccine groups (1–5) with Tukey's HSD post hoc test.

Compared Mouse Groups		IFN-γ	TNF-α	IL-10	IFN-γ/IL-10	TNF-α/IL-10	SI
**Ag (rPfCelTOS)**	**Ag/CpG**	**0.002***	**0.011***	0.985	0.227	0.959	0.112
	**Ag/Poly I:C**	**0.007***	0.057	0.987	0.591	0.999	0.34
	**Ag/CpG+Poly I:C**	**<0.0001*****	**0.001****	0.913	**<0.0001*****	0.757	**<0.0001*****
	**Ag/CFA/IFA**	0.504	0.543	0.894	0.068	0.977	0.964
**Ag/CpG**	**Ag/Poly I:C**	0.966	0.869	1.000	0.98	0.999	0.978
	**Ag/CpG+Poly I:C**	**<0.0001*****	0.206	1.000	**0.003***	0.999	**0.001****
	**Ag/CFA/IFA**	**0.024***	0.132	1.000	**0.003***	0.576	0.376
**Ag/Poly I:C**	**Ag/CpG+Poly I:C**	**<0.0001*****	**0.039***	1.000	**0.001****	0.942	**0.001****
	**Ag/CFA/IFA**	0.081	0.595	1.000	0.007	0.845	0.819
**Ag/CpG+Poly I:C**	**Ag/CFA/IFA**	**<0.0001*****	**0.004***	1.000	**<0.0001*****	0.323	**<0.0001*****

$P < 0.05$ were considered statistically significant and shown with star(s) and bold (*$P < 0.05$, **$P \leq 0.001$, ***$P < 0.0001$).

TIgG total IgG
