# Supplementary material for: Cell-traversal protein for ookinetes and sporozoites (CelTOS) formulated with potent TLR adjuvants induces high-affinity antibodies that inhibit Plasmodium falciparum infection in Anopheles stephensi
Source: Malar J. 2019 Apr 24;18:146. doi: 10.1186/s12936-019-2773-3 (PMC6480871; doi:10.1186/s12936-019-2773-3)
Supplement: Supplementary file 2 — Additional file 2: Table S1. Multiple comparisons of means anti-rPfCelTOS IgG, its subclasses, Th1/Th2 ratio and anti-rPfCelTOS avidity antibodies among the non-adjuvanted (group 1) and adjuvanted (groups 2—5) vaccine groups on day 38 of the first immunization using Tukey’s HSD post hoc test. Table S2. Multiple comparisons of mean IFN-γ, TNF, and IL-10 cytokines levels, IFN-γ/IL-10 and TNF/IL-10 ratios, and stimulation Index (SI) of MTT assay among all vaccine groups (1—5) with Tukey’s HSD post hoc test. Table S3. Effect of anti-rPfCelTOS IgG antibodies induced in mice on P. falciparum infectivity in An. stephensi. [file 12936_2019_2773_MOESM2_ESM.zip › Additional file2 Tables/Additional file 2 Table S3.pdf]

Additional file 2: Table S3 Effect of anti-rPfCelTOS IgG antibodies induced in mice on *P. falciparum* infectivity in *An. stephensi*.

	**Antibody**	**Arithmetic mean no. of oocysts in midgut (range)**	**No. of infected/dissected mosquito (prevalence %)**
**Vaccine Groups**	Ag	4.4 (0-11)	42/49 (85.7)
	Ag/CpG	2.5 (0-8)	32/40 (80)
	Ag/Poly I:C	2.2 (0-8)	31/41 (75.6)
	Ag/CpG+ Poly I:C	1.8 (0-6)	41/51 (80.4)
**Control Groups**	NMS	8.3 (0-35)	53/61 (86.9)
	CpG	8 (0-34)	50/59 (84.7)
	Poly I:C	8.1 (0-33)	52/60 (86.7)
	CpG+Poly I:C	7.9 (0-33)	51/60 (85)

Pooled sera from negative control groups (NMS, non-adjuvanted control group; CpG, Poly I:C, and CpG + Poly I:C, adjuvanted control groups) and from different vaccinated groups were used in SMFA. Statistical analysis of mean oocyst numbers per midgut in all different control groups showed no significant difference ($P > 0.05$) by using Mann-Whitney *U*-test. The pooled anti-rPfCelTOS antibodies from all the vaccine groups significantly inhibited oocyst formation in *An. stephensi* relative to the NMS control group ($P < 0.05$, Mann-Whitney *U*-test).

Ag rPfCelTOS antigen

NMS normal mouse sera
